# *Leishmania* regulates host YY1: Comparative proteomic analysis identifies infection modulated YY1 dependent proteins

**DOI:** 10.1371/journal.pone.0323227

**Published:** 2025-05-15

**Authors:** Harsimran Kaur Brar, Eleanor Chen, Fabian Chang, Shawna Angel Lu, Dilraj Kaur Longowal, Kyung-Mee Moon, Leonard J. Foster, Neil Reiner, Devki Nandan

**Affiliations:** 1 Division of Infectious Diseases, Department of Medicine, University of British Columbia, Vancouver, British Columbia, Canada; 2 Department of Biochemistry and Molecular Biology, University of British Columbia, Vancouver, British Columbia, Canada; Universita degli Studi della Campania Luigi Vanvitelli, ITALY

## Abstract

The protein Yin-Yang 1 (YY1) is a ubiquitous multifunctional transcription factor. Interestingly, there are several cellular functions controlled by YY1 that could play a role in *Leishmania* pathogenesis. Leishmaniasis is a human disease caused by protozoan parasites of the genus *Leishmania.* This study examined the potential role of macrophage YY1 in promoting *Leishmania* intracellular survival. Deliberate knockdown of YY1 resulted in attenuated survival of *Leishmania* in infected macrophages, suggesting a role of YY1 in *Leishmania* persistence. Biochemical fractionation studies revealed *Leishmania* infection caused redistribution of YY1 to the cytoplasm from the nucleus where it is primarily located. Inhibition of nuclear transport by leptomycin B attenuates infection-mediated YY1 redistribution and reduces *Leishmania* survival. This suggests that *Leishmania* induces the translocation of YY1 from the nucleus to the cytoplasm of infected cells, where it may regulate host molecules to favour parasite survival. A label-free quantitative whole proteome approach showed that the expression of a large number of macrophage proteins was dependent on the YY1 level. Interestingly, several of these proteins were modulated in *Leishmania*-infected cells, revealing YY1-dependent host response and suggesting their potential role in *Leishmania* pathogenesis. Together, this study identifies YY1 as a novel virulence factor that promotes *Leishmania* survival inside host macrophages.

## Introduction

Leishmaniasis is a vector-borne disease caused by the intracellular protozoan parasite of the genus *Leishmania* [1,2]. The life cycle of *Leishmania* alternates between two major different cell morphologies. The motile promastigote morphology exists in the lumen of the sandfly where as non-motile amastigote morphology resides in the phagolysosome of mammalian mononuclear phagocytes [3]. In humans, leishmaniasis has two main clinical forms, cutaneous leishmaniasis and visceral leishmaniasis. Visceral leishmaniasis, the more severe form of the disease, is mainly caused by infection with *Leishmania donovani* [1] and is most likely fatal if left untreated. According to WHO, an estimated 50,000–90,000 new cases of visceral leishmaniasis occur worldwide each year [4]. Unfortunately, no vaccines are available against any forms of leishmaniasis in humans, only a few approved vaccines are registered for canine leishmaniasis [5]. Additionally, current therapies are toxic to healthy cells and have limited efficacy [6]. More in-depth knowledge regarding *Leishmania-*host macrophage interactions may lead to the development of new innovative therapies to cure and control leishmaniasis.

Yin-Yang 1 (YY1) is a ubiquitous, multifunctional, zinc-finger transcription factor that regulates genes directly by binding to gene promoters or indirectly by interacting with other transcription factors [7,8]. Interestingly, through transcriptomic profiling, YY1 has been identified as one of three transcriptional regulators activated by *Leishmania* amastigotes in bone marrow derived macrophages [9]. This may serve as an initial hint towards YY1’s possible involvement in promoting *Leishmania* intracellular survival specifically. Moreover, YY1 has been implicated in the control of various cellular mechanisms that likely benefit *Leishmania* pathogenesis. For example, YY1 has been linked to anti-inflammatory M2 macrophage polarization [10,11]. This would create a pro-parasitic, immunosuppressive environment that would enable *Leishmania* survival [12,13]. Additionally, YY1 has been shown to interact with and activate cell signaling protein Akt [14] in breast cancer cells. Interestingly, *Leishmania* infection has also been shown to activate Akt, which enhances parasite survival in host macrophages by inhibiting apoptosis and pro-inflammatory responses. In fact, Akt inhibition directly resulted in a marked decrease in parasite survival [15].

Although initially discovered as a nuclear transcription factor [16], YY1 has since been found to be present in the cytoplasm of several cultured cell lines [17–19] and tissues [19,20], which may suggest alternative, non-transcriptional roles of YY1. In this context, it was interesting to note that vaccinia virus induces YY1 translocation from the nucleus to the cytoplasm of infected human macrophages, where it seems to take part in pathogenesis [21,22]. This raised the possibility that *Leishmania* could induce translocation of YY1 to the cytoplasm to engage YY1 interaction with cytoplasmic factor in infected cells to its favor.

In this study, we show that host macrophage YY1 plays an important role in the persistence of *Leishmania* in infected cells. The deliberate knockdown of YY1 significantly attenuated the survival of *Leishmania* in human macrophages, demonstrating that YY1 is essential for *Leishmania* persistence. Strikingly, *Leishmania* redirects YY1 from its primary location of the nucleus to the cytoplasm during infection. Treatment with leptomycin B, an inhibitor of the Crm1 transport system, blocked YY1 transport to the cytoplasm and attenuated *Leishmania* survival. Comparative quantitative proteomic analysis revealed that the expression of a large number of macrophage proteins (537 out of 6559 proteins) was dependent on the level of YY1. Interestingly, the expression of many YY1-dependent proteins was affected in *Leishmania*-infected cells, suggesting the role of YY1 in *Leishmania* pathogenesis. Taken together, this study identifies host macrophage YY1 as a novel virulence factor that *Leishmania* regulates to promote its survival in infected cells*.*

## Materials and methods

### Ethics statement

Buffy coats from healthy donors were purchased from the Canadian Blood Services Network Centre for Applied Development Ethics (Institutional Ethics Certificate: H23-00636). The source of human blood cells did not disclose the identity of the human donors, thereby anonymizing all data to the investigators. This study abides by the principles of the Declaration of Helsinki. All work with animals in this study was reviewed and approved by the University of British Columbia Animal Care Committee (protocol license number A14-0218). The animal care and use of protocol adhered to the standards and regulations provided by the Canadian Council on Animal Care in Science.

### Reagents and antibodies

The human monocytic cell line, THP- 1 (TIB-202TM), was purchased from American Type Culture Collection (ATCC). RPMI 1640 media and Fetal bovine serum (FBS) were obtained from Gibco. HEPES, penicillin/streptomycin, L-glutamine, Hanks’ balanced salt solution (HBSS), M199 medium, folic acid, hemin, adenosine, and Phorbol 12-myristate 13-acetate (PMA) were purchased from Millipore Sigma. Anti-Lamin A/C (#2032), anti-YY1 (D5D9Z), and leptomycin B (#9676) were purchased from Cell Signaling. Anti-actin (SC-47778) was obtained from Santa Cruz biotechnology. Anti-GAPDH (G041) was obtained from Applied Biological Materials Inc.

### Culturing and differentiation of THP-1 cells

THP-1 cells were cultured in RPMI 1640 medium supplemented with 10% heat-inactivated FBS, 10 mM HEPES, 100 U/mL penicillin/streptomycin, and 2 mM L-glutamine at 37^0^C with 5% CO_2_. Cells were passaged every 2–3 days. To induce differentiation, THP-1 cells were treated with 10 ng/mL PMA for 16–18 hours. Adhered differentiated THP-1 (dTHP-1) cells were then subjected to washings with HBSS and provided with fresh media lacking PMA. The cells were then rested for 24 hours before any subsequent treatment.

### Parasite culture and infection of dTHP-1 cells

*Leishmania donovani* Sudan strain S2 promastigotes were cultured in M199 medium supplemented with 10% heat-inactivated FBS, 20 mM HEPES, 10 μg/mL folic acid, 3 μg/mL hemin, 2 mM L-glutamine, 100 U/mL penicillin/streptomycin, and 100 μM adenosine at 26°C. Virulence was maintained by regular passages in Syrian Golden hamsters from which fresh amastigotes were obtained from heavily infected hamaster spleen tissue essentially as described [23] and *in vitro* transformed into promastigotes as previously described [24]. For *in vitro* infection, stationary phase promastigotes were incubated with dTHP-1 cells at a MOI (*Leishmania*: THP-1) of 20:1 unless otherwise indicated. Infection rate determination was performed as previously described [24].

### Purification of human monocytes and *in vitro* differentiation to macrophages

Buffy coats from healthy donors were purchased from Canadian Blood Services Network Centre and diluted 1:2 in PBS supplemented with EDTA. Peripheral blood mononuclear cells (PBMCs) were isolated from buffy coat using density gradient centrifugation on Ficoll-Paque PLUS (Cytiva) at room temperature. After isolation, cells were washed with PBS to remove platelets. The cells were then allowed to adhere in a 5% CO_2_ incubator at 37°C for 1 hour. Non‐adherent cells were removed by thorough washings with RPMI 1640. Adherent cells were collected using a cell scraper, and monocytes were counted using Tuerk’s solution. The cells were plated at a density of 0.75 × 10^6^ cells/mL in multiwell plates in RPMI medium supplemented with 10% FBS, 2 mM L-glutamine, and 100 U/mL penicillin/streptomycin. For monocyte derived macrophage (hMDM) differentiation, purified cells were plated in cell culture dishes in complete maturation media (RPMI 1640 with 10% FCS, 100 U/mL penicillin/streptomycin, and 10 ng/mL granulocytes-macrophage colony-stimulating factor (GM‐CSF) (Peprotech, Stockholm, Sweden). After 3 days, the cells were washed with HBSS and given fresh supplemented RPMI medium containing GM-CSF. The cells were washed again on day 6, rested in supplemented RPMI medium without GM–CSF, and used for experiments on day 7.

### siRNA transfection

3 unique YY1 (human) siRNA duplexes and one non-specific (scrambled) negative control were obtained from OriGene (SR322232). THP-1 and hMDMs Macrophages were transfected with 100 nM of siRNA using the HiPerfect (Qiagen) transfection reagent according to the manufacturer’s protocol. The plates were gently mixed to ensure uniform distribution of the transfection complex. THP-1 cells were treated with siRNA 24 hours prior to PMA-mediated differentiation and hMDMs were treated with siRNA for 24 hours after GM-CSF-mediated differentiation. To confirm YY1 knockdown, whole cell lysates of transfected cells were analysed for YY1 levels by Western blotting 24 hours post transfection. YY1 deficient cells were subsequently incubated with or without *Leishmania* and assessed in downstream assays.

### Western blotting

Cells were washed with HBSS and lysed in ice-cold cell lysis buffer (20 mM Tris-HCl, pH 7.4, 1% Triton X-100, 1 mM EDTA, 0.15 M NaCl, 1 mM sodium orthovanadate, 5 mM NaF, 5 μg/mL aprotinin, 5 μg/mL leupeptin, and 2 mM PMSF) on ice. For *L. donovani* whole cell lysates, parasites were washed with HBSS and lysed in ice-cold cell lysis buffer with double the aprotinin, leupeptin, and PMSF concentration. Lysates were boiled in equal volumes of 4X Laemmli sample loading buffer for 7 min, separated by SDS-PAGE and transferred to nitrocellulose membrane using a semi-dry transfer apparatus. Transferred proteins were probed with appropriate antibodies, according to the manufacturer’s instructions. Protein bands were captured on Blue X-ray film (Carestream) using ECL Select. Densitometry analysis was performed using ImageJ.

### Parasite rescue assay

dTHP-1 cells or hMDMs were infected with stationary phase *L. donovani* at a MOI of 20:1. After 24 hours of infection, cells were extensively washed to remove non-internalized *Leishmania*. Infected cells were then lysed using 0.01% SDS, followed by the transformation of live, rescued *Leishmania* amastigotes to promastigotes in M199 media by incubating plates at 26°C for 48 hours. The evaluation of their growth was performed by manual counting of transformed promastigotes using a hemocytometer as described previously [24–26].

### Biochemical subcellular fractionation

Subcellular fractionation was performed as previously described [27] with minor modifications. Briefly, THP-1 cells (1.5 x 10^6^ cells per well) were differentiated in a six-well plate with PMA. Cells were lysed in 900 μL of digitonin lysis buffer (50 mM HEPES pH 7.4, 150 mM NaCl, 20 μg/mL digitonin, 1 mM PMSF, 5 μg/mL aprotinin, 5 μg/mL leupeptin). Lysates were incubated on ice for 10 minutes with intermittent mixing followed by centrifugation at 2000 x g for 4 minutes at 4°C. The supernatant was carefully removed and the remaining cell pellet was extracted in 2X SDS-PAGE sample loading buffer. Extracted proteins from each fraction were analyzed by Western blot using an anti-GAPDH antibody as a marker of the cytosol and an anti-Lamin A/C as markers of the nucleus.

### Liquid chromatography – tandem mass spectrometry and protein identification

Cell pellets were lysed in 50% (v/v) 2,2,2-trifluoroethanol, reduced and alkylated, and digested with trypsin [28]. The peptides were cleaned with C-18 STop And Go Extraction (STAGE) tips [29] and eluted using 40% (v/v) acetonitrile in 0.1% (v/v) formic acid. The peptides were resuspended in Buffer A consisting of 0.5% acetonitrile, 0.1% formic acid in MS grade water and peptide concentration was estimated by UV absorbance at 205nm using a NanoDropOne (Thermo Scientific, A205nm scopes). Approximately 100 ng of peptides were loaded onto timsTOF Pro2 (Bruker Daltonics) coupled to NanoElute UHPLC (Bruker Daltonics) using Aurora Series Gen3 25 cm analytical column (IonOpticks) using a DIA-PASEF method. Instrument parameters follow paper [30] with following method changes. The PASEF ramps contained 25 isolation windows from 299.5 m/z to 1200.5 m/z with ion mobility range of 0.7 - 1.3 V ⋅ s ⋅ cm^-2^ and collision energy ramped from 20.0 eV at 0.6 V⋅s⋅cm^-2^ to 65.0 eV at 1.6 V⋅s⋅cm^-2^. Sample injection order was randomized.

Data were searched against Uniprot’s human and *Leishmania donovani* proteomes (UP000005640 and UP000008980) with a custom list of contaminants using DIA-NN version 1.9.1 [31]. Library-free search mode was enabled, using trypsin/P protease specificity and 1 missed cleavages. Other search parameters include 1 maximum number of variable modification, N-terminal M excision, carbamidomethylation of C, and oxidation of M. Peptide length ranged 7–30, precursor charge ranged 2–4, precursor and fragment m/z ranged 200–1700. Precursor FDR was set to 1%, with 0 for settings ‘mass accuracy’, ‘MS1 accuracy’ and ‘scan window’. Settings ‘heuristic protein inference’, ‘match between run (MBR)’, and ‘no shared spectra’ were all enabled. ‘Protein name from FASTA’ was chosen for protein inference parameter along with ‘double-pass mode’ for neural network classifier. ‘QuantUMS (high precision)’ was used for quantification strategy, ‘RT-dependent’ mode for cross-run normalisation, and ‘IDs, RT & IM profiling’ mode for library generation.

The mass spectrometry proteomics data have been deposited to the ProteomeXchange Consortium via the PRIDE [32] partner repository with the dataset identifier PXD059968.

### Quantitative analysis of liquid chromatography-tandem mass spectrometry data

All downstream analysis was carried out in Microsoft Excel and R software (http://www.r-project.org). From the total number of proteins detected by liquid chromatography-tandem mass spectrometry (LC-MS/MS), potential contaminants and *L. donovani* proteins were filtered out. Only proteins that had raw intensity values in all three replicates were retained for further analysis. All downstream analyses were performed on log2-transformed ratios (fold changes). A two-sample, two-tailed T-test was conducted to compare the log2-transformed normalized ratios. A p-value of less than 0.05 was considered statistically significant. Benjamini-Hochberg procedure was used to further calculate adjusted p-values [33,34]. No minimum fold change cut-off was set for a protein to be considered modulated. Volcano plot, comparing average log_2_ values with -log10(p-value) was also plotted using R software for better visualization.

Uniprot Princeton GO Mapper (https://go.princeton.edu/cgi-bin/GOTermMapper) was used for Gene Ontology (GO) annotation with the following considerations: Organism annotation set to “Homo sapiens (GOA @EBI + Ensembl)” and Ontology set to “Generic slim.” The protein annotation involved three biological aspects: Biological process (“process”), Molecular function (“function”), and Cellular component (“component”). GO terms with three or more protein members were kept. Additionally, broad terms like “Organelle” spanning several subcategories were omitted; for identical GO terms, only one term was retained (e.g., “Extracellular space” was kept, while “Extracellular region” was removed; “Extracellular matrix” was retained while “External encapsulating structure” was omitted; “Carbohydrate derivative metabolic process” was kept and “Carbohydrate metabolic process” was omitted; “Mitotic nuclear division” was omitted while keeping “Mitotic cell cycle”; “Catalytic activity” was kept, while “Catalytic activity acting on proteins or DNA” was omitted).

## Results

### YY1 protein is required for the survival of *Leishmania* in infected dTHP-1 cells

**Fig 1:**
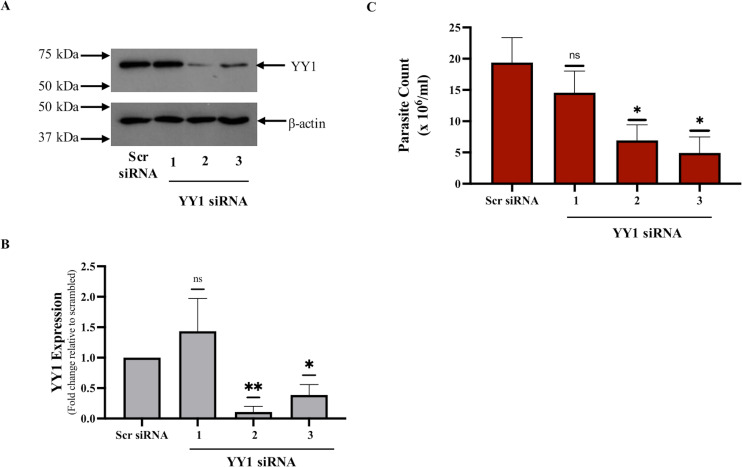
YY1 knockdown inhibits *L. donovani* survival in dTHP-1 cells. (A) THP-1 cells were transfected with three unique YY1 siRNAs or a scrambled siRNA (scr siRNA) for 24 hours followed by differentiation with PMA, and subsequently infection with *L. donovani* at a 20:1 MOI for 24 hours. Cells were washed extensively and Western blotting was performed using the specified antibodies on whole-cell lysates of infected dTHP-1 cells. (B) Histogram representing densitometric analysis of YY1 knockdown normalized to Actin levels. Bars represent mean ± SD of three independent experiments. (C) Parasite rescue assay was performed in parallel as described in “Materials and methods”. Bars represent the mean ± SD of four independent experiments. Statistical significance was determined using two-sample two-tailed T-test (ns: not significant, *: p < 0.05, **: p < 0.01).

To investigate the possible role of host macrophage YY1 protein in the perseverance of *Leishmania*, we downregulated YY1 in THP-1 cells using siRNA mediated gene silencing and assessed parasite survival. Effectiveness of YY1 knockdown in THP-1 cells was determined by Western blot analysis on whole cell lysates (Fig 1A and 1B). Two of the three YY1 siRNAs used were successful in significantly knocking down YY1 protein levels compared to the control siRNA and were selected for use in downstream experiments. To analyze the effects of YY1 knockdown on *Leishmania* survival, control and YY1 knockdown cells were differentiated with PMA for 24 hours post siRNA transfection, infected with *L. donovani* for 24 hours, then assessed using a parasite rescue assay as described in “Materials and methods”. The results of the parasite rescue assay, presented in Fig 1C, show a clear reduction of *Leishmania* survival in YY1 knockdown cells. Together, our data strongly suggest that macrophage YY1 could play an important role in the perseverance of *Leishmania* in infected host cells.

### YY1 is critical for *Leishmania* survival in primary human macrophages

**Fig 2:**
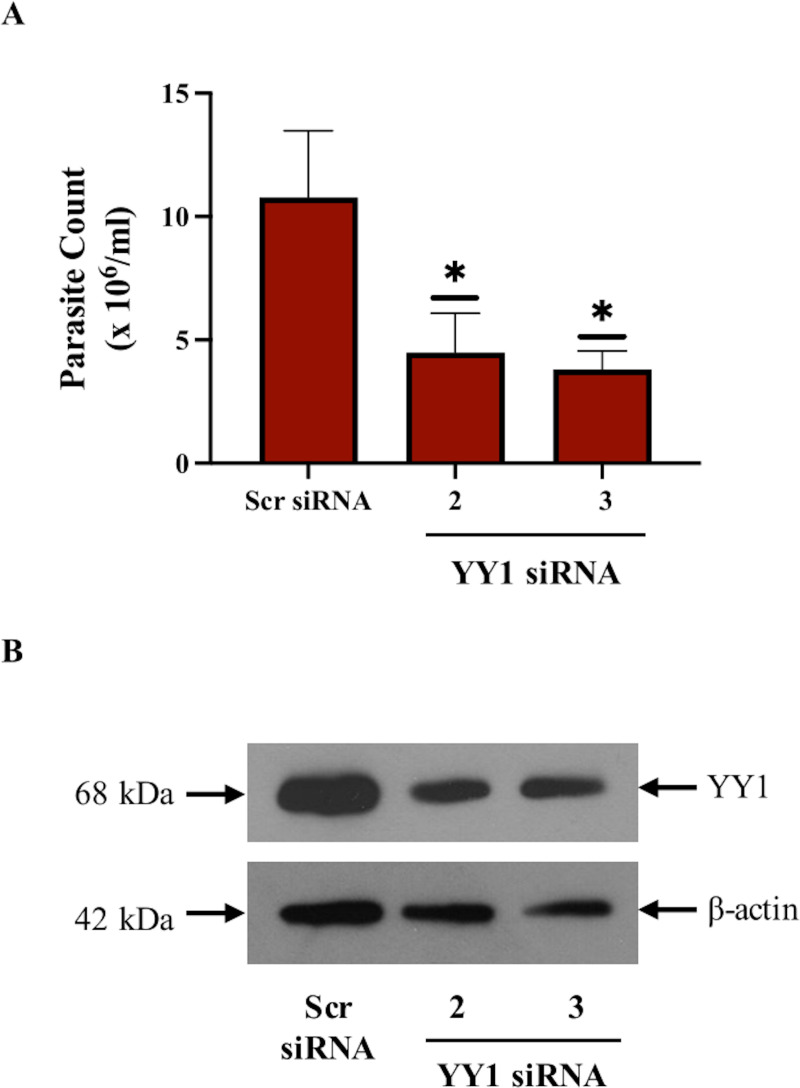
YY1 knockdown reduces *L. donovani* survival in hMDMs. (A) Peripheral human blood monocytes were isolated and differentiated to macrophages as described in “Materials and methods”. hMDMs were transfected with two unique YY1 siRNAs or a scrambled siRNA (scr siRNA) for 24 h followed by infection with *L. donovani* at a 20:1 MOI for 24 h. The parasite rescue assay was then performed as described in “Materials and methods”. Bars represent mean ± SD of three independent experiments. Statistical significance was determined using two-sample two-tailed T-test (*: p < 0.05). (B) YY1 knockdown was verified by Western blot using the indicated antibodies on whole cell lysates of infected hMDMs.

The results presented above in Fig 1 showed that YY1 is required for the survival of *Leishmania* in infected dTHP-1 cells. While THP-1 cells are widely used in leishmaniasis related studies [35–38], it was of interest to validate results presented in Fig 1 in primary human macrophages. Therefore, peripheral human blood monocytes were isolated from healthy donors and differentiated into macrophages (hMDMs) to investigate the role of YY1 in *Leishmania* survival in these cells. For this investigation, YY1 was downregulated in hMDMs by transfecting with two different siRNAs against YY1. A scrambled siRNA was used as a control. After 24 hours of transfection, cells were infected with *L.donovani*. 24 hours post infection, cells were lysed and processed for a parasite rescue assay as described in “Materials and methods”. In parallel, cells were also processed for Western Blot analysis to ensure effectiveness of siRNAs to knockdown YY1 (Fig 2B). The results shown in Fig 2A clearly demonstrate that YY1 is important for the survival of *Leishmania* in hMDMs thus validating the results obtained using dTHP-1 cells.

### *Leishmania* mediates cytoplasmic translocation of YY1 in infected host cells

Working with human primary human monocytes/macrophages, in contrast to macrophage/monocytic cell lines, has limitations such as limited yield from a single donor, inter donor variability, shorter lifespan, limited expansion capacity, and notoriously difficult transfection. To counter these limitations, rest of the study presented here was performed in PMA diffentiated THP-1 cells.

Despite YY1 being a transcription factor, it has been found in both the nucleus and the cytoplasm of cultured cell lines [18,19]. This raises the possibility that cytoplasmic YY1 may have functions unrelated to its primary role as a transcription factor. In the context of host-pathogen interactions, a previous study showed that vaccinia virus mediates Crm 1-dependent cytoplasmic translocation of YY1 in infected cells [22]. However, viral infection did not alter YY1 levels at the whole cell level [22]. Given that *Leishmania* survival requires YY1 expression in host macrophages, *Leishmania* infection may modulate YY1 expression. To investigate whether *Leishmania* alters the abundance of host YY1, dTHP-1 cells were infected with *L. donovani* (MOI 10:1 and 20:1) and YY1 protein levels were monitored by Western blotting over 48 hours. As shown in supplementary Fig S1, expression of YY1 over 48 hours of infection at both MOIs was not significantly affected relative to non-infected control cells.

**Fig 3:**
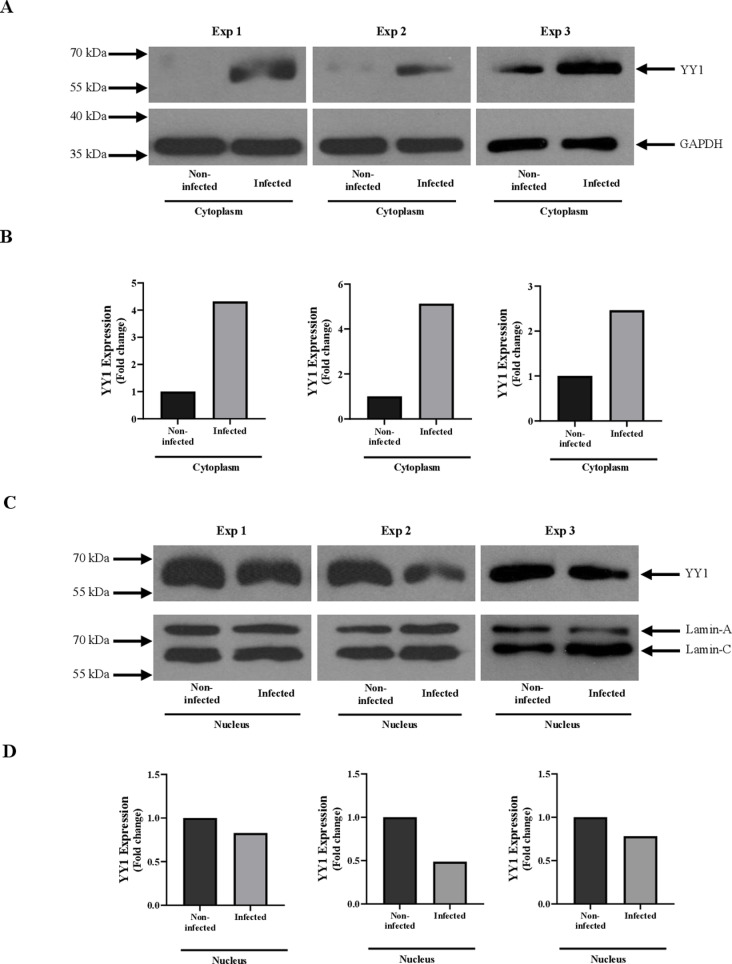
*L. donovani* infection induces cytoplasmic translocation of YY1 in dTHP-1 cells. dTHP-1 cells were infected with *L. donovani* at 20:1 MOI for 24 hours and cellular fractionation was performed as described in “Materials and methods”. (A) The cytoplasmic fractions of non-infected and infected cells were then Western blotted using the indicated antibodies. (B) Histograms representing densitometric analysis of Western blots of cytoplasmic fractions from dTHP-1 cells. (C) The nuclear fractions of non-infected and infected cells were Western blotted using the indicated antibodies. (D) Histograms representing densitometric analysis of western blots of nuclear fractions from dTHP-1 cells. The data shown are from three independent experiments.

Since we didn’t see any significant changes in YY1 expression at whole cell level, it was of interest to investigate whether like vaccinia virus, *Leishmania* infection also induces cytoplasmic translocation of host macrophage YY1 from its primary location in the nucleus. The intracellular localization of YY1 during *Leishmania* infection in THP-1 was investigated by performing biochemical cell fractionation as described in “Material and methods”. Cells were gently lysed using digitonin and nuclei were pelleted by centrifugation. Lamin A/C and GAPDH were used as specific markers of the nuclear and the cytoplasmic fractions, respectively. As shown in Fig 3A and 3C, GAPDH and Lamin A/C were highly enriched in cytoplasmic and nuclear fractions, respectively. Cytoplasmic fractions were also tested for the possible contamination of Lamin A/C (Fig S2). The results, presented in Fig 3, show that *Leishmania* causes a substantial increase in cytoplasmic YY1 levels, while reducing nuclear YY1 levels in infected THP-1 cells. This suggests that *Leishmania* induces YY1 translocation to the cytoplasm from the nucleus during infection.

### *Leishmania* mediated cytoplasmic transport of host YY1 protein involves Crm 1 transport system

**Fig 4:**
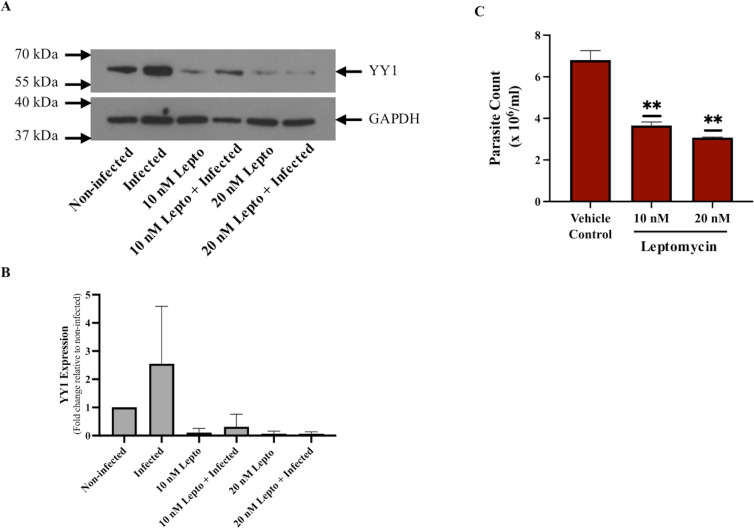
Leptomycin B inhibits cytoplasmic translocation of YY1 in dTHP-1 cells. (A) dTHP-1 cells were either untreated or treated with leptomycin B (Lepto) at a concentration of 10 nM or 20 nM for 3 hours prior to infection with *L. donovani* at a MOI of 20:1. After 24 hours, cells were fractionated as described in ”Materials and methods”. YY1 protein levels in cytoplasmic fractions were verified by Western blotting with the indicated antibodies. (B) Histogram representing densitometric analysis of YY1 expression in Western blot of cytoplasmic fractions from dTHP-1 cells. (C) **Leptomycin B attenuates *L. donovani* survival in dTHP-1 cells**. In parallel, parasite rescue assay was performed as described in “Materials and methods”. For this assay, dTHP-1 cells were either treated with vehicle alone, or treated with leptomycin B at a concentration of 10 nM or 20 nM for 3 hours, followed by infection with *L. donovani* at a MOI of 20:1 for 24 hours. Bars represent the mean ± SD of three independent experiments. Statistical significance was determined using two-sample two-tailed T-test (**: p < 0.01).

The observed increase in cytoplasmic YY1 level may be due to increased YY1 nuclear export or decreased nuclear import. To differentiate between the two mechanisms, we selectively inhibited the nuclear export of proteins. Classic nuclear export occurs through the nuclear pore complexes involving exportins (also known as chromosomal maintenance 1, Crm1) [39]. In the absence of a characterized nuclear export signal of YY1, we used leptomycin B to inhibit Crm1 mediated nuclear export. Leptomycin B, initially used as an anti-fungal agent, was the first specific inhibitor of the Crm1 system reported [40]. For this study, dTHP-1 cells were pretreated with leptomycin B then washed and followed by *Leishmania* infection for 24 hours. Cytosolic fractions from non-infected and infected cells were analyzed for the presence of YY1 by Western blotting. As shown in Fig 4A and 4B, treatment of cells with leptomycin B resulted in decreased cytoplasmic YY1 level in both control and *Leishmania* infection conditions. This suggests that the main origin of cytoplasmic YY1 is from the export of nuclear YY1; *Leishmania* likely increases YY1 nuclear export via the Crm1 export system rather than increasing cytoplasmic retention of de novo cytoplasmic YY1 or decreasing nuclear import. In parallel, to confirm that cytoplasmic YY1 is important for *Leishmania* survival, we performed a parasite rescue assay in leptomycin B treated cells. As expected, blockage of nuclear export by leptomycin B significantly attenuated survival of *Leishmania* in infected cells as compared to vehicle alone treated infected cells (Fig 4C).

The results thus far show that YY1 is translocated to cytoplasm during *Leishmania* infection, and host macrophage YY1 is important for optimal intracellular survival of *Leishmania*. These interesting findings prompted us to further study the role of YY1 in the pathogenesis of *Leishmania* infection. To this end, we used a comprehensive, unbiased label free quantitative proteomic analysis of *Leishmania*-infected cells in an YY1-deficient condition.

### Comparative quantitative proteomic analysis of *Leishmania*-infected macrophages

**Fig 5:**
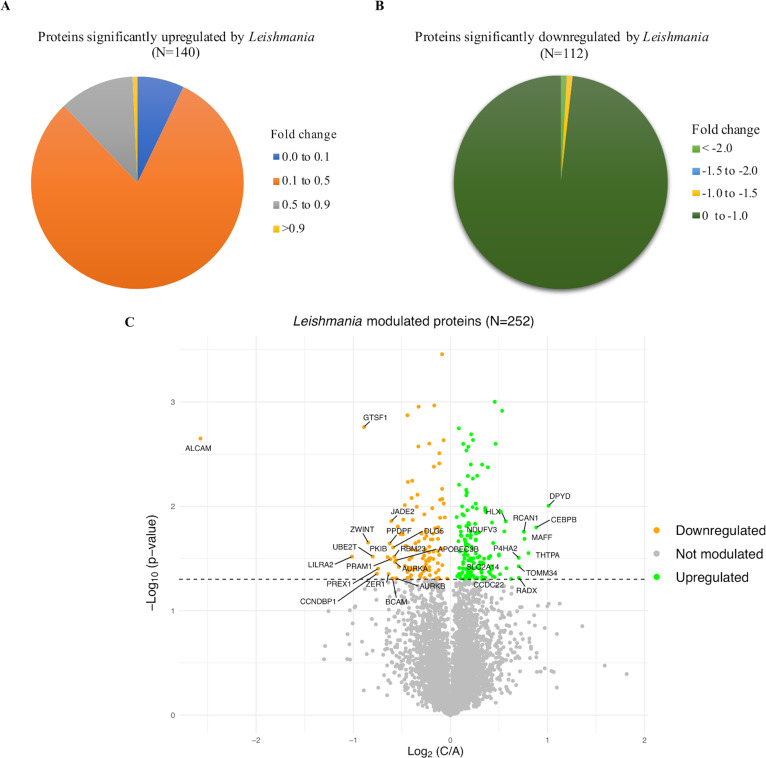
Comparative analysis of global proteome of control and *L. donovani* infected cells. dTHP-1 cells were either infected with *L. donovani* or not. Cells were then dislodged in cell dissociation buffer (without enzyme) and mass spectrometric analysis was perfomed as described in “Materials and methods”. (A) Pie-chart depicting the distribution of proteins significantly upregulated by *L. donovani* infection, categorized based on the average of log2 values from three independent replicates (fold change). (B) Pie-chart depicting the distribution of proteins significantly downregulated by *L. donovani* infection, categorizd based on the average of log2 values from three independent experiments. Two sample, two tailed T-test was used to measure statistical significance with p-value <0.05 considered significant. (C) Volcano plot of proteins showing significant fold changes in *L. donovani* infected cells as compared to non-infected cells. Proteins significantly upregulated by *L. donovani* infection are shown in green dots, those that were downregulated, as orange dots and not modulated as grey dots. Data is from three independent experiments. The horizontal dashed line represents the p-value cut off of 0.05 or -log10(p-value) of 1.301. **C** denotes infected; **A** denotes non-infected.

The aim of the study was to investigate YY1 dependent host proteome during *L. donovani* infection. Therefore, it was important to first determine how *L. donovani* alters the proteome of dTHP-1 cells. For this, quantitative proteomic analysis was done for non-infected and infected dTHP-1 cells. We first filtered out the common contaminants and *L. donovani* proteins from initial list of 14220 proteins identified. This resulted in total of 10487 proteins. For downstream analysis, only those proteins that had raw intensity values in all three biological replicates in both uninfected and infected cells were used (6585 proteins) (Table S1). 252/6585 proteins were found to be significantly modulated (adjusted p-value < 0.05) by *L. donovani*, out of which 140 proteins were upregulated and 112 were downregulated (Table S1) (Fig 5A and 5B). Volcano plot which illustrates the *Leishmania* modulated proteins (upregulated/downregulated) has also been plotted (Fig 5C) for better visualization of the data.

### Identification of host macrophage proteins modulated by *Leishmania* and dependent on YY1

**Fig 6:**
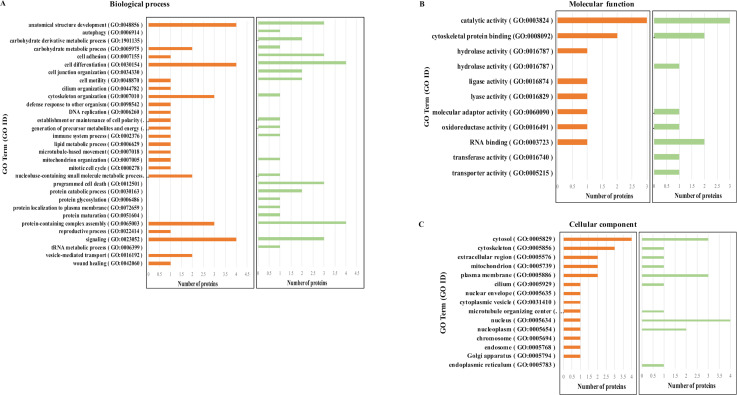
Gene ontology analysis of YY1-dependent *L. donovani* modulated proteins in THP-1 cells. Gene ontology (GO) enrichment analysis was performed on significantly modulated proteins by *L. donovani* (upregulated or downregulated) and recovered by YY1 (downregulated and upregulated, respectively) knockdown. Proteins were categorized into: (A) Biological process, (B) Molecular Function, and (C) Cellular component. Orange represents the proteins significantly upregulated by *L. donovani* and recovered by YY1 and green represents the proteins significantly downregulated by *L. donovani* and recovered by YY1.

For this study, we performed a quantitative proteomic analysis of *L.donovani*-infected cells in a YY1-deficient condition and compared it to proteome of *L.donovani*-infected normal cells. We chose those proteins that have raw intensity values in all replicates of control and *L. donovani* infected and YY1 knockdown and *L. donovani* infected cells, which resulted in a total of 245 proteins (Table S2). Next, we choose (i) proteins upregulated in *L. donovani* infected cells (average log2(C/A) > 0), and that were recovered closer to non-infected abundance, (average log2 (D/C) < 0) by YY1 knockdown, and (ii) proteins downregulated by *L. donovani* infection (average log2 (C/A) < 0) and were recovered by YY1 knockdown (average log2 (D/C) > 0) (Table 1). In average log2 analysis, A represents protein level in control and uninfected cells; C represents protein level in control and *L. donovani* infected cells and D represents protein level in YY1 knockdown and *L. donovani* infected cells. 31/245 proteins were observed to be significantly modulated by YY1 knockdown in *L. donovani*-infected cells. Of these, 16 proteins were recovered (8 proteins upregulated, 8 proteins downregulated) by YY1 knockdown (Table 1; Table S2). The *L. donovani* infection sensitive and YY1 dependent host proteins were also classified by GO terms in three groups: biological process, molecular function, and cellular component (Fig 6A–6C).

**Table 1:** YY1-dependent *L. donovani*-modulated host proteins in dTHP-1 cells.

*YY1-dependent L. donovani- upregulated host proteins in dTHP-1 cells*				
Protein description	Gene name	Uniprot ID	Fold change (Average Log_2 _C/A > 0)	Fold change (Average Log_2 _D/C < 0)
Tubulin polyglutamylase complex subunit 1 (PGs1)	TPGS1 C19orf20	Q6ZTW0	0.429287	-1.022591511
Pleckstrin (Platelet 47 kDa protein) (p47)	PLEK P47	P08567	0.193316	-0.02834847
FERM domain-containing protein 4A	FRMD4A FRMD4 KIAA1294	Q9P2Q2	0.264011	-0.550649734
Dihydropyrimidinase-related protein 2 (DRP-2) (Collapsin response mediator protein 2) (CRMP-2) (N2A3) (Unc-33-like phosphoprotein 2) (ULIP-2)	DPYSL2 CRMP2 ULIP2	Q16555	0.224368	-0.406896493
Cytochrome c oxidase assembly protein COX19 (hCOX19)	COX19	Q49B96	0.20478	-0.567578851
Golgi integral membrane protein 4 (Golgi integral membrane protein, cis) (GIMPc) (Golgi phosphoprotein 4) (Golgi-localized phosphoprotein of 130 kDa) (Golgi phosphoprotein of 130 kDa)	GOLIM4 GIMPC GOLPH4 GPP130	O00461	0.145495	-0.227740876
Ankyrin repeat domain-containing protein 17 (Gene trap ankyrin repeat protein) (Serologically defined breast cancer antigen NY-BR-16)	ANKRD17 GTAR KIAA0697	O75179	0.192595	-0.211788204
NADP-dependent malic enzyme (NADP-ME) (EC 1.1.1.40) (Malic enzyme 1)	ME1	P48163	0.181688	-0.367545834

*YY1-dependent L. donovani- downregulated host proteins in dTHP-1 cells*				
Protein description	Gene names	Uniprot ID	Fold change (Average Log_2 _C/A < 0)	Fold change (Average Log_2 _D/C > 0)
Schlafen family member 5	SLFN5	Q08AF3	-0.10537	0.487793
tRNA (34–2’-O)-methyltransferase regulator WDR6 (WD repeat-containing protein 6)	WDR6	Q9NNW5	-0.34629	0.391072
Tripartite motif-containing protein 2 (EC 2.3.2.27) (E3 ubiquitin-protein ligase TRIM2) (RING finger protein 86) (RING-type E3 ubiquitin transferase TRIM2)	TRIM2 KIAA0517 RNF86	Q9C040	-0.26802	0.408278
Afadin (ALL1-fused gene from chromosome 6 protein) (Protein AF-6) (Afadin adherens junction formation factor)	AFDN AF6 MLLT4	P55196	-0.10618	0.458748
ER degradation-enhancing alpha-mannosidase-like protein 3 (EC 3.2.1.113) (Alpha-1,2-mannosidase EDEM3)	EDEM3 C1orf22	Q9BZQ6	-0.10487	0.300969
Disks large homolog 5 (Discs large protein P-dlg) (Placenta and prostate DLG)	DLG5 KIAA0583 PDLG	Q8TDM6	-0.59043	0.272367
Cytoplasmic FMR1-interacting protein 2 (p53-inducible protein 121)	CYFIP2 KIAA1168 PIR121	Q96F07	-0.22064	0.184068
NADH dehydrogenase [ubiquinone] 1 beta subcomplex subunit 6 (Complex I-B17) (CI-B17) (NADH-ubiquinone oxidoreductase B17 subunit)	NDUFB6	O95139	-0.43758	0.313633

### Global proteome analysis of YY1 knockdown THP-1 cells and control THP-1 cells

**Fig 7:**
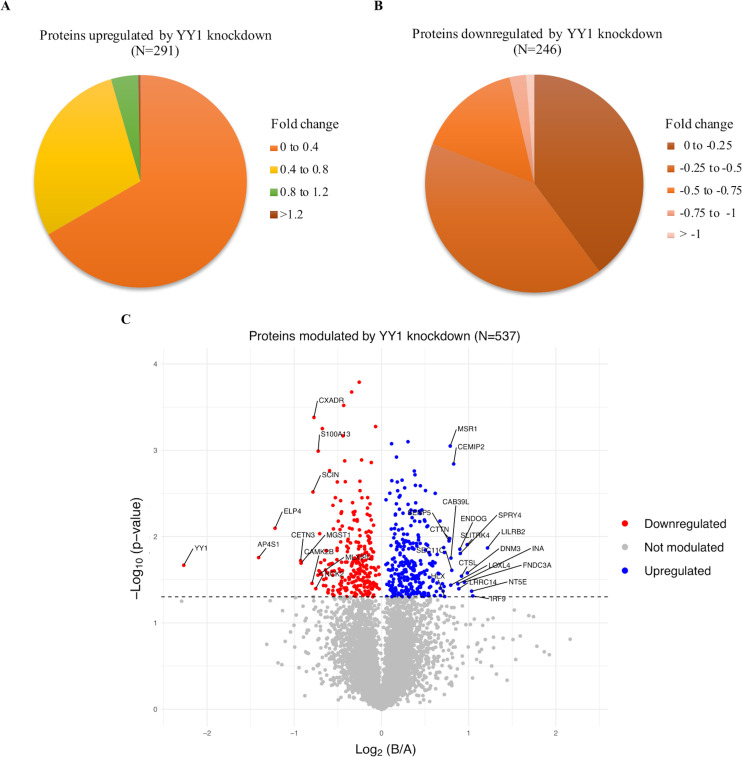
Comparative analysis of global proteome of control and YY1 knockdown cells. THP-1 cells were transfected with equal mix of two different siRNAs (50 nM each) or a scrambled siRNA for 24 hours, followed by differentiation. Cells were then dislodged in cell dissociation solution (without enzyme) and mass spectrometric analysis was perfomed as described in “Materials and methods”. (A) Pie-chart illustrating the distribution of proteins significantly upregulated by YY1 knockdown, categorized based on average of log2 values. (B) Pie-chart demonstrating the distribution of proteins significantly downregulated by YY1 knockdown, categorized based on average of log2 values. Two sample, two tailed T-test was used to measure statistical significance with p-value <0.05 considered significant. (C) Volcano plot of proteins showing significant fold changes in YY1 knockdown cells as compared to control cells. Proteins significantly upregulated by YY1 knockdown are shown as blue dots, those that were downregulated, as red dots and those that are not modulated as grey dots. Data is from three independent experiments. The p-value cut off of 0.05 or - log10(p-value) of 1.301 is represented by the horizontal dashed line. **B** denotes YY1 knockdown macrophages; **A** denotes control macrophages.

It is becoming increasingly clear that YY1 is a ubiquitous protein that serves many important and diverse biological roles [7,41]. Expectedly, our proteomic results presented in Table S3 revealed that a large number of macrophage proteins (approximately 5% of identified proteins) are sensitive to abundance of YY1. An extensive literature search revealed no published global protein profiling of YY1 dependent proteins in THP-1 cells or other macrophages in humans or murine models. Thus, it was of interest to analyse YY1-sensitive macrophage proteins in detail. For this analysis, proteins that showed raw intensity values in all three replicates in both control and YY1 knockdown macrophages were chosen (6559 proteins) (Table S3). Out of the 6559 proteins, 537 showed significant changes (adjusted p-value < 0.05) in abundance in the YY1 knockdown cells. Of the 537 proteins significantly modulated, expression level of 291 proteins increased (Fig 7A) while expression of 246 decreased in the YY1 knockdown THP-1 cells (Fig 7B) (Table S3). As seen in Table 3, YY1 is the most significantly downregulated protein which validates our knockdown experiment. The global proteome changes, in YY1 knockdown THP-1 cells were also visualized in a volcano plot as shown in Fig 7C.

### Gene ontology analysis of YY1 sensitive macrophage proteins

**Fig 8:**
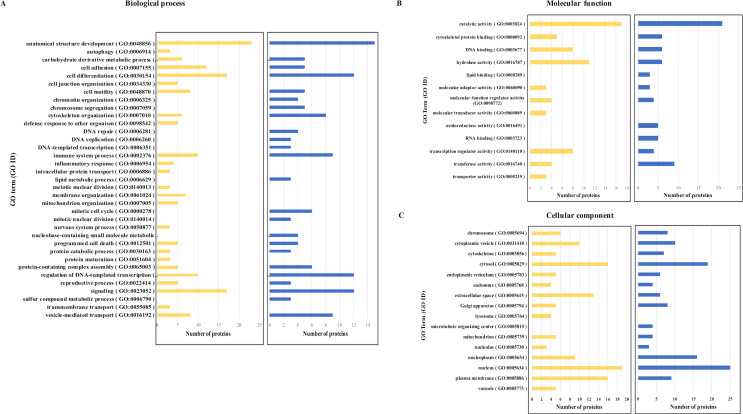
Gene ontology analysis of significantly modulated proteins in YY1 knockdown dTHP-1 cells. Gene ontology (GO) enrichment analysis was performed on top 30 most significantly upregulated and downregulated proteins in YY1 knockdown dTHP-1 cells and classified into three categories: (A) Biological process, (B) Molecular function, and (C) Cellular component. Yellow represents the proteins upregulated by YY1 knockdown and blue represents the proteins downregulated by YY1 knockdown.

To further analyze YY1 sensitive macrophage proteins regarding their biological importance, all significantly modulated proteins were organized into three categories by GO terms: biological process, molecular function and cellular component (Tables **S4–S6** in File S1). However, the top 30 proteins upregulated or downregulated significantly by YY1 knockdown are presented in Table 2 and 3 along with their GO terms in three categories: biological process, molecular function and cellular component (Fig 8).

**Table 2:** Top 30 proteins upregulated by YY1 knockdown in dTHP-1 cells.

Protein description	Uniprot ID	Gene name	Fold change (Average log_2_)	p-value	Adjusted p-value	Known roles related to pathogens	Known roles in Immunity
Leukocyte immunoglobulin-like receptor subfamily B member 2	Q8N423	LILRB2	1.21638239	0.013567783	0.013780261	Inhibitory receptor LILRB2 is targeted for immune evasion of *P. falciparum* [42] Infection with *Salmonella typhimurium* led to an upregulation of LILRB2 [43]	Transmit inhibitory signals in macrophages and other myeloid cells [44]
Interferon regulatory factor 9	Q00978	IRF9	1.046978197	0.04869277	0.048696462	NA	IRF9, a transcription factor, facilitates the expression of interferon-stimulated genes through JAK-STAT pathway [45]
5’-nucleotidase	P21589	NT5E	1.034390315	0.042954619	0.043389199	NA	NT5E performs numerous homeostatic functions in healthy organs and tissues [46]
Dynamin-3	Q9UQ16	DNM3	0.984937977	0.02642286	0.027560521	NA	NA
SLIT and NTRK-like protein 4	Q8IW52	SLITRK4	0.983317362	0.012409657	0.012569832	NA	NA
Fibronectin type-III domain-containing protein 3A	Q9Y2H6	FNDC3A	0.950278077	0.033766848	0.034450652	NA	NA
Procathepsin L	P07711	CTSL	0.918165432	0.028752389	0.030167598	Ctsl helps contain mycoplasma infection by supporting host immune response to infection [47]	NA
Protein sprouty homolog 4	Q9C004	SPRY4	0.90391386	0.015701084	0.016014898	NA	NA
Endonuclease G, mitochondrial	Q14249	ENDOG	0.89919109	0.014025527	0.014711359	*Leptospira interrogans* induces apoptosis in macrophages by mediating mitochondrial release of AIF and/or EndoG via Bid [48] Herpes simplex virus (HSV) uses host UL12.5 to deploy host cellular proteins, including ENDOG, to destroy mtDNA for their survival [49]	EndoG, a mitochondrion-specific nuclease is involved in caspase-independent apoptotic pathway [50]
Leucine-rich repeat-containing protein 14	Q15048	LRRC14	0.887431751	0.040263633	0.040689013	NA	LRRC14 is a negative regulator of TLR signaling, which is important for innate immunity [51]
Alpha-internexin	Q16352	INA	0.875746072	0.035352176	0.03594041	NA	NA
Cell surface hyaluronidase	Q9UHN6	CEMIP2	0.827603099	0.001443178	0.001489758	NA	NA
Signal peptidase complex catalytic subunit SEC11C	Q9BY50	SEC11C	0.806515055	0.024626403	0.025325885	NA	NA
Calcium-binding protein 39-like	Q9H9S4	CAB39L	0.796717849	0.01777717	0.017225326	NA	NA
Lysyl oxidase homolog 4	Q96JB6	LOXL4	0.794796243	0.036592403	0.037057728	*C. parvum* RNA enters host nuclei upon infection and causes suppression of the cadherin 3 (CDH3) gene in human intestinal epithelial cells which is involved in cell migration and adherence [52]	NA
Macrophage scavenger receptor types I and II	P21757	MSR1	0.788720284	0.000893981	0.000931099	MSR1 was required for TLR3-mediated recognition of the extracellular HCV dsRNA [53]	Involved in M2 macrophage polarization; component of other pathogen pattern recognition receptor signaling complexes [54]
Src substrate cortactin	Q14247	CTTN	0.782302917	0.010545141	0.009962756	Cortactin tyrosine phosphorylation is important for invasion of *C. parvum* in biliary epithelial cells [55]	NA
Sentrin-specific protease 5	Q96HI0	SENP5	0.774620406	0.011258233	0.010707635	NA	NA
H2.0-like homeobox protein	Q14774	HLX	0.72500883	0.049567595	0.049627561	NA	Functions in supporting normal hematopoietic cell proliferation [56] Drives maturation of Th1 and IFN-γ secretion in cooperation with T-bet [57]
Bcl-2-like protein 11	O43521	BCL2L11	0.722370217	0.015296611	0.015735568	Deletion of BCL2L11 in *L. major* infected mice significantly increased CD4 + T-cell responses and were resistant to persistent infection [58]	Promotes intrinsic pathway of apoptosis [59]. Its absence promotes autoimmunity.
Transcription factor Sp1	P08047	SP1	0.718280004	0.037904517	0.038081937	Host SP1 was involved in maintaining transcription of HSV-1 genes in fibroblasts [60]. SP1-mediates transcriptional regulation of *TINAGL1* expression in gastric epithelial cells (GECs) during *H. pylori* infection, ensuring *H. pylori* persistence and gastritis [61].	Basal transcription factor, involved in regulation of housekeeping genes [62] SP1 is also known to bind YY1 transcription factor [63]. They both have binding sites on promoter regions of various genes [64].
Zinc finger protein 561	Q8N587	ZNF561	0.713728075	0.013418262	0.013687151	NA	NA
Osteopontin	P10451	SPP1	0.706695852	0.04288259	0.043296089	*H. pylori* infection in gastric cancer cells induces SPP1 activation, leading to T cell inactivation [65]	Identified as a cytokine (Eta-1) which is produced by activated T cells [66]
Thymidine phosphorylase	P19971	TYMP	0.699170573	0.035217122	0.0358473		Plays an important role in the pyrimidine salvage pathway [67]
F-box only protein 6	Q9NRD1	FBXO6	0.675798441	0.048262757	0.048044693	Impairs the survival of alveolar macrophages and antiviral immunity of the host in Inflenza A virus infection[68] Modulates IFN-I-mediated antiviral immune responses [69]	F-box family of proteins are the key components of SKP1-Cullin1-F-box (SCF) E3 ligase and controls cell cycle, cell proliferation and cell death [69]
Metal cation symporter ZIP14	Q15043	SLC39A14	0.674353222	0.039646534	0.040223464	NA	NA
Protein spinster homolog 1	Q9H2V7	SPNS1	0.670739943	0.006613296	0.00679702	NA	NA
RUN and FYVE domain-containing protein 2	Q8WXA3	RUFY2	0.658920147	0.026968881	0.028026071	NA	NA
Squalene monooxygenase	Q14534	SQLE	0.649560533	0.026601845	0.027746741	NA	NA
N-acetylglucosamine-6-sulfatase	P15586	GNS	0.639485243	0.016125673	0.016108007	NA	NA

**Table 3:** Top 30 proteins downregulated by YY1 knockdown in dTHP-1 cells.

Protein description	Uniprot ID	Gene name	Fold change (Average log_2_)	p-value	Adjusted p-value	Known roles related to pathogens	Known roles in Immunity
Transcriptional repressor protein YY1	P25490	YY1	-2.267320196	0.021510917	0.022346369	YY1 binds to the promoter of TLR4, promoting its transcription, and aggravating the proinflammatory response of macrophages infected with MTB [70]	NA
AP-4 complex subunit sigma-1	Q9Y587	AP4S1	-1.408672747	0.01752392	0.016945996	NA	NA
Elongator complex protein 4	Q96EB1	ELP4	-1.220185205	0.008009702	0.007914339	NA	NA
Centrin-3	O15182	CETN3	-0.926879521	0.019029529	0.018528864	NA	NA
Microsomal glutathione S-transferase 1	P10620	MGST1	-0.921717382	0.020366426	0.020577281	NA	NA
Calcium/calmodulin-dependent protein kinase type II subunit beta	Q13554	CAMK2B	-0.796009973	0.03487625	0.03556797	NA	NA
Scinderin	Q9Y6U3	SCIN	-0.785144146	0.003042603	0.002979516	NA	NA
Coxsackievirus and adenovirus receptor	P78310	CXADR	-0.774148618	0.00041697	0.000372439	NA	The deletion of the Macrophage-Specific Coxsackievirus and Adenovirus Receptor in mice enhances M1 polarity and myocarditis in Coxsackievirus B3 infection [71]
Cytoplasmic protein NCK2	O43639	NCK2	-0.754010333	0.040158869	0.040595903	NA	NA
Carbohydrate-responsive element-binding protein	Q9NP71	MLXIPL	-0.728503229	0.027999148	0.029329609	NA	NA
Protein S100-A13	Q99584	S100A13	-0.725199313	0.001024552	0.001024209	NA	NA
Ribonucleoside-diphosphate reductase large subunit	P23921	RRM1	-0.717153352	0.024875109	0.025698324	NA	NA
Synaptotagmin-like protein 1	Q8IYJ3	SYTL1	-0.708352904	0.009257719	0.009031657	NA	Synaptotagmin-like protein 1 has a role in exocytosis of secretory lysosomes from Cytotoxic T cells [72]
CMP-N-acetylneuraminate-beta-galactosamide-alpha-2,3-sialyltransferase 4	Q11206	ST3GAL4	-0.702765513	0.025815532	0.026815642	NA	NA
C-type mannose receptor 2	Q9UBG0	MRC2	-0.696165189	0.020003412	0.020018622	NA	NA
Tubulin polyglutamylase complex subunit 1	Q6ZTW0	TPGS1	-0.691959047	0.027312439	0.02867784	NA	NA
12S rRNA N4-methylcytidine (m4C) methyltransferase	A6NJ78	METTL15	-0.67807795	0.000561304	0.000558659	NA	NA
VPS35 endosomal protein-sorting factor-like	Q7Z3J2	VPS35L	-0.664751721	0.036921255	0.037430168	NA	NA
Calcineurin B homologous protein 3	Q96BS2	TESC	-0.661603623	0.031456749	0.032588454	NA	NA
Vacuolar protein sorting-associated protein 26C	O14972	VPS26C	-0.653308776	0.018758228	0.018063315	NA	NA
Core histone macro-H2A.2	Q9P0M6	MACROH2A2	-0.641820049	0.024358876	0.024767225	NA	NA
Esterase OVCA2	Q8WZ82	OVCA2	-0.638708916	0.037142379	0.037616387	NA	NA
Thiamin pyrophosphokinase 1	Q9H3S4	TPK1	-0.635451369	0.030548762	0.031843575	NA	NA
Mitotic spindle assembly checkpoint protein MAD2A	Q13257	MAD2L1	-0.635171686	0.014560012	0.015176909	NA	NA
60S ribosomal protein L35	P42766	RPL35	-0.622559748	0.026383389	0.027374302	NA	NA
Golgin subfamily A member 8O	F8WBI6; A6NCC3; H3BV12; I6L899	GOLGA8N;GOLGA8O;GOLGA8Q;GOLGA8R	-0.614271427	0.043977852	0.044227188	NA	NA
Leucine-rich repeat-containing protein 58	Q96CX6	LRRC58	-0.612591557	0.044858458	0.044972067	NA	NA
5’-nucleotidase domain-containing protein 1	Q5TFE4	NT5DC1	-0.608545779	0.041379186	0.041806331	NA	NA
Proteasome assembly chaperone 2	Q969U7	PSMG2	-0.603988015	0.021100345	0.02150838	NA	NA
Neuroserpin	Q99574	SERPINI1	-0.595052875	0.001725209	0.001582868	NA	NA

Regarding biological functions associated with altered proteins in YY1 knockdown cells, the highest number of proteins upregulated and downregulated by YY1 knockdown were associated with anatomical structural development, cell differentiation, and signaling (Fig 8A). A high number of proteins downregulated by YY1 knockdown were also seen involved in the regulation of DNA-templated transcription. Proteins affected by YY1 knockdown were also involved in programmed cell death (Fig 8A). In the case of molecular function, both upregulated and downregulated proteins were mostly involved in catalytic activity (Fig 8B). In the case of cellular localization, most of the proteins downregulated or upregulated were seen to be present in both the nucleus and cytoplasm (Fig 8C), but highest in nucleus as can be expected from YY1 being a transcriptional factor. In the case of upregulated proteins, a high number of proteins were also seen to be part of the plasma membrane.

It was intriguing to investigate the significance of YY1-dependent macrophage proteins beyond leishmaniasis, particularly in the context of infection and immunity. For this purpose, we reviewed the literature related to infection and immunity, primarily utilizing PubMed, Scopus, and Google Scholar biological databases. In this search, we excluded Covid-19. Of the 537 YY1-dependent proteins, 54 have previously been reported in infections and 50 in immunity-related studies. Interestingly, proteins that are upregulated by YY1 knockdown are more involved in infection and immunity (Table S3).

## Discussion

In the current study, we identified YY1 as a novel virulence factor promoting *Leishmania* survival in our macrophage-cell model dTHP-1 and primary human monocyte derived macrophages. Thus depleting endogenous YY1 resulted in significantly reduced survival of *Leishmania* in infected cells. YY1 is a transcriptional factor ubiquitously expressed in humans and is known to have a fundamental role in several normal biological processes via its ability to initiate, activate, or repress transcription depending upon the context in which it binds [16,73,74]. Interestingly, some of the known functions of YY1 such as being antiapoptotic [75–77] and interaction with cell signaling proteins Akt [14] are relevant to the pathogenesis of *Leishmania* infection [15,78]. The above properties of YY1 were the basis of our hypothesis that *Leishmania* regulates host YY1 to promote its survival in infected host cells, leading us to initiate the current study. Based on this presumptive role of YY1 in *Leishmania* survival, we expected that the knockdown of YY1 in macrophages would impact *Leishmania* survival. In the present study, siRNAs against YY1 successfully knocked down expression of YY1 in both dTHP-1 and hMDMs, as confirmed by Western blot analysis (Figs 1A and 2B). Control cells and YY1 knockdown cells were infected for parasite rescue assay. Here, we observed a significant decrease in the survival of *Leishmania* as compared to infected control cells, demonstrating that YY1 plays an important role in *Leishmania* survival within host cells (Figs 1C and 2A).

In addition to YY1 presence in the nucleus, its localization in the cytoplasm has also been observed. The presence of YY1 in both the nucleus and the cytoplasm [17–20] suggests a role of YY1 other than as a transcription factor [74]. Although the transcriptional activities and gene regulatory functions of nuclear YY1 is well described, the information regarding the function of YY1 in the cytoplasm is very limited. The redirection of YY1 localization from the nucleus to the cytoplasm has been shown in BSC-40 cells and human blood adherent monocytes in response to vaccinia virus infection [21,22] suggesting that vaccinia virus has evolved to recruit cellular YY1 for viral gene expression. The virus mediated translocation of YY1 seems to be dependent on Crm1 transport system. In fact, the Crm1-dependent export system is used by many viral proteins: HIV-1 Rev protein, adenovirus E4 34 kDa protein and hepatitis B virus proteins [79]. Our result presented in this study shows that translocation of nuclear YY1 to the cytoplasm seems to be dependent on the Crm1 export system [22] since leptomycin B treatment greatly diminished the level of cytoplasmic YY1. As shown in Fig 3, *Leishmania*, like vaccinia virus, induced redirection of YY1 from the nucleus to the cytoplasm of infected macrophages using a Crm1-dependent export system. As expected, pre-treatment of cells with leptomycin B decreased survival of *Leishmania* in infected cells, strongly suggesting that transport of YY1 to the cytoplasm could be required for the optimal survival of *Leishmania.*

At present, we do not know the function of cytoplasmic YY1 in *Leishmania* infected cells. However, decreased survival of *Leishmania* found upon leptomycin B treatment suggests potential role of cytoplasmic YY1 in *Leishmania* survival. Nevertheless, our result suggests that in human macrophages infected with *Leishmania*, the Crm1 system is involved in YY1 transport to the cytoplasm. Identification of YY1 interacting factors in the cytoplasmic fraction of infected macrophages has potential to shed light on the cytoplasmic function of YY1 in pathogenesis of *Leishmania* infection. Perhaps, this translocation is required to induce interaction of YY1 to host factors to create a pro-parasitic environment suitable for *Leishmania* survival

To further elucidate the role of YY1 in *Leishmania* persistence, we performed comparative proteomic analysis of *Leishmania* infected YY1 knockdown cells. This analysis revealed differentially expressed proteins during *Leishmania* infection that are dependent on YY1. Interestingly, none of these YY1 dependent proteins has been implicated in *Leishmania* infection -related processes in previously published studies. However, further investigation of *Leishmania-*modulated YY1 dependent proteins is highly desirable. GO terms analysis of *Leishmania* upregulated YY1 dependent proteins revealed top GO terms for biological process as signaling (GO:0023052), cell differentiation (GO:0030154) anatomical structure development (GO:0048856). GO terms analysis of *Leishmania* downregulated YY1 dependent proteins revealed top GO terms for biological process as cell differentiation (GO:0030154) and protein-containing complex assembly (GO:0065003).

Given that YY1 knockdown results in broad effects on the host proteomic profile (approximately 5% of total identified proteins were affected), it was of interest to classify these YY1 sensitive proteins by GO terms in three categories: biological process, molecular function and cellular components. The top 10 GO terms for proteins that are upregulated by YY1 knockdown in the biological process category include anatomical structure development (GO:0048856), signaling (GO:0023052), cell differentiation (GO:0030154), regulation of DNA-templated transcription (GO:0006355), immune system process (GO:0002376), vesicle-mediated transport (GO:0016192), programmed cell death (GO:0012501), cell adhesion (GO:0007155), transmembrane transport (GO:0055085), protein-containing complex assembly (GO:0065003), and cytoskeleton organization (GO:0007010). This was not surprising as YY1 is known to participate in various biological functions including apoptosis, cell prolifertion, differentiation, replication and embryogenesis [7,74]. Our analysis also showed that top 10 GO terms in biological process category related to YY1 dependent downregulated proteins are anatomical structure development (GO:0048856), signaling (GO:0023052), cell differentiation (GO:0030154) regulation of DNA-templated transcription (GO:0006355), vesicle-mediated transport (GO:0016192), cytoskeleton organization (GO:0007010), mitotic cell cycle (GO:0000278), protein-containing complex assembly (GO:0065003), and programmed cell death (GO:0012501).

Additionally, we analyzed the possible role of YY1 modulated proteins in infections other than leishmaniasis and in immunity. Our literature search relevant to infection and immunity revealed that out of 291 upregulated proteins, 43 have been implicated in infections, and 41 in immunity. Furthermore, of the 246 downregulated proteins, 11 are previously known to be associated with infection and 9 with immunity (Table S3). Together, the quantitative proteomic analysis of YY1 knockdown cells revealed that a large number of YY1 sensitive proteins are related to infection and immunity based on published reports. How many of the remaining YY1 sensitive macrophage proteins are related to infection and imunity remains to be investigated. We do note the limitations of our proteomic study, specifically the use of a single monocytic cell line, THP-1, and the absence of a broader and more extensive evaluation of the proteome data. Moreover, we understand that our data is limited to proteomic analysis, and it will be of interest to support these findings with relevant biological assays, warranting future studies. Nevertheless, proteomic data presented here can be a valuable resource for other researchers interested in YY1 functions and its role in infection and immunity.

In summary, the present study demonstrates that macrophage YY1 is required for the optimal survival of *Leishmania* in infected cells. It was also observed that *Leishmania* redirected nuclear YY1 to the cytoplasm during infection. Perhaps, this translocation is required to induce interaction with cytoplasmic factor to create a pro-parasitic environment suitable for *Leishmania* survival. Taken together, we have identified YY1 as a novel and essential protein that promotes *Leishmania* survival. To our knowledge, this is the first report showing the role of host YY1 in the pathogenesis of an intracellular parasitic infection and may have implications for other intracellular pathogens. In addition, to our knowledge, this is the first report providing global landscape of proteome-wide alterations that occur in human macrophages after knockdown of YY1. PMA-differentiated THP-1 cells have been extensively used as a model system to study macrophage-pathogen interactions involving intracellular pathogens such as *Leishmania* [35,36,80]*, Mycobacterium tuberculosis* [81,82], *Toxoplasma gondii* [83] and HIV [84,85]. THP-1 has also been used as macrophage-cell model to study monocyte/macrophage functions [37,86]. Thus, YY1 sensitive proteome analysis of dTHP-1 is expected to provide valuable information regarding biological functions of human YY1 at least in macrophages.

## Supporting information

Fig S1 *L. donovani* infection does not alter YY1 protein abundance in dTHP-1 cells. A) dTHP-1 cells were infected with *L. donovani* at a 10:1 or 20:1 MOI for 4, 10, 24, or 48 hours. The whole cell lysates from non-infected and *L. donovani*-infected cells were Western blotted for the indicated antibodies. B) Histogram of the densitometric analysis of whole cell lysates in Western blots. Bars represent the mean ± SD of three independent experiments. Statistical significance was determined using two-sample two tailed T-test; h.p.i refers to hours post-infection.
(TIF)

Fig S2 Lamin A/C are not detected in cytoplasmic fractions of non-infected and *L. donovani* infected dTHP-1 cells. A) The cytoplasmic fraction of non-infected and *Leishmania*-infected dTHP-1 cells and the nuclear fraction of non-infected dTHP-1 cells were analyzed by Western blot for the nuclear marker Lamin A/C (n = 3).
(TIFF)

Table S1 Proteins significantly modulated by *L. donovani* infection in dTHP-1 cells. (XLSX)

Table S2 Proteins significantly modulated by *L. donovani* and recovered by YY1 knockdown in dTHP-1 cells. (XLSX)

Table S3 Proteins significantly modulated by YY1 knockdown in dTHP-1 cells. (XLSX)

File S1 Tables S4-S6. Gene ontology analysis of significantly modulated proteins in YY1 knockdown dTHP-1 cells. Table S4 represents biological process, Table S5 represents molecular function, and Table S6 represents cellular component.
(DOCX)

S1 File Raw images. (PDF)
