# Supplementary figures and images for: Leishmania regulates host YY1: Comparative proteomic analysis identifies infection modulated YY1 dependent proteins
Source: PLoS One. 2025 May 15;20(5):e0323227. doi: 10.1371/journal.pone.0323227 (PMC12080872; doi:10.1371/journal.pone.0323227)

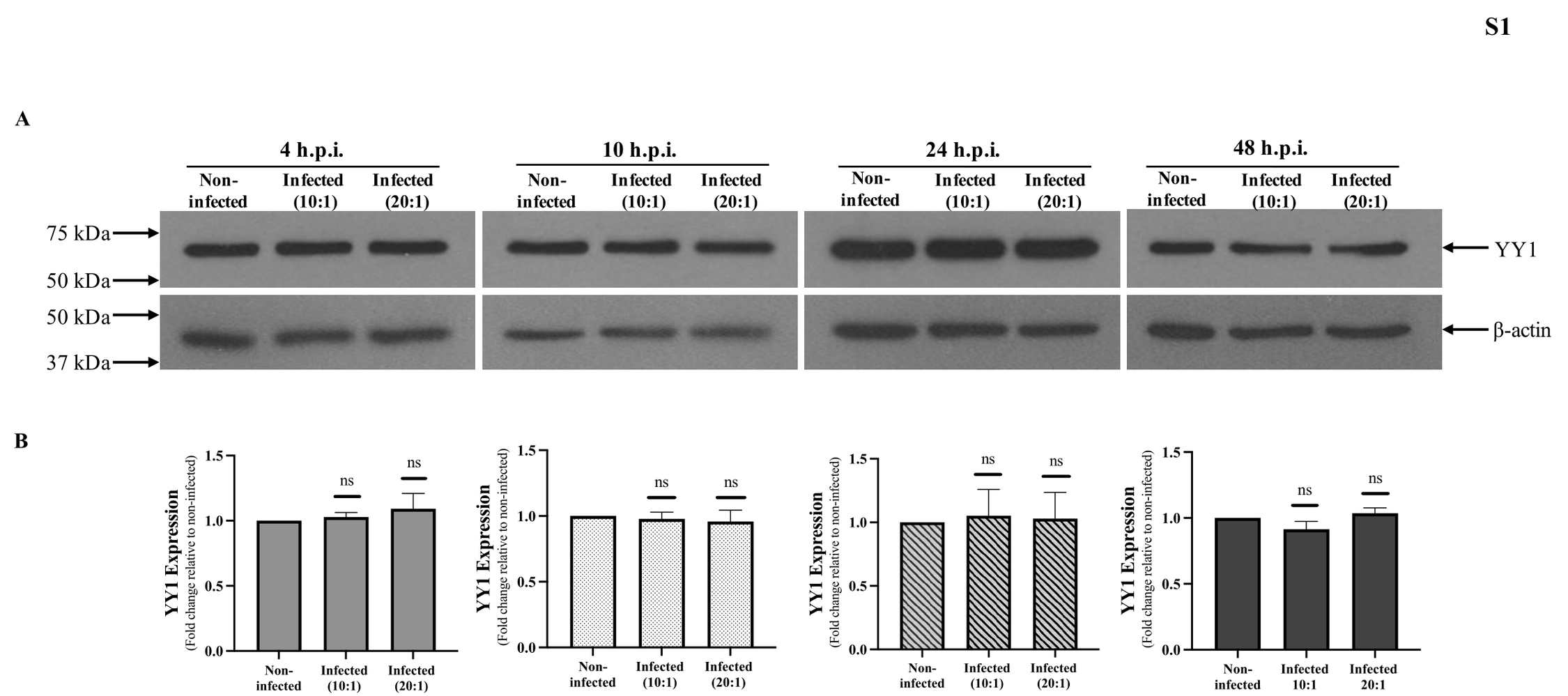

Supplement: Fig S1 — A) dTHP-1 cells were infected with L. donovani at a 10:1 or 20:1 MOI for 4, 10, 24, or 48 hours. The whole cell lysates from non-infected and L. donovani-infected cells were Western blotted for the indicated antibodies. B) Histogram of the densitometric analysis of whole cell lysates in Western blots. Bars represent the mean ± SD of three independent experiments. Statistical significance was determined using two-sample two tailed T-test; h.p.i refers to hours post-infection. (TIF) [file pone.0323227.s001.tif]

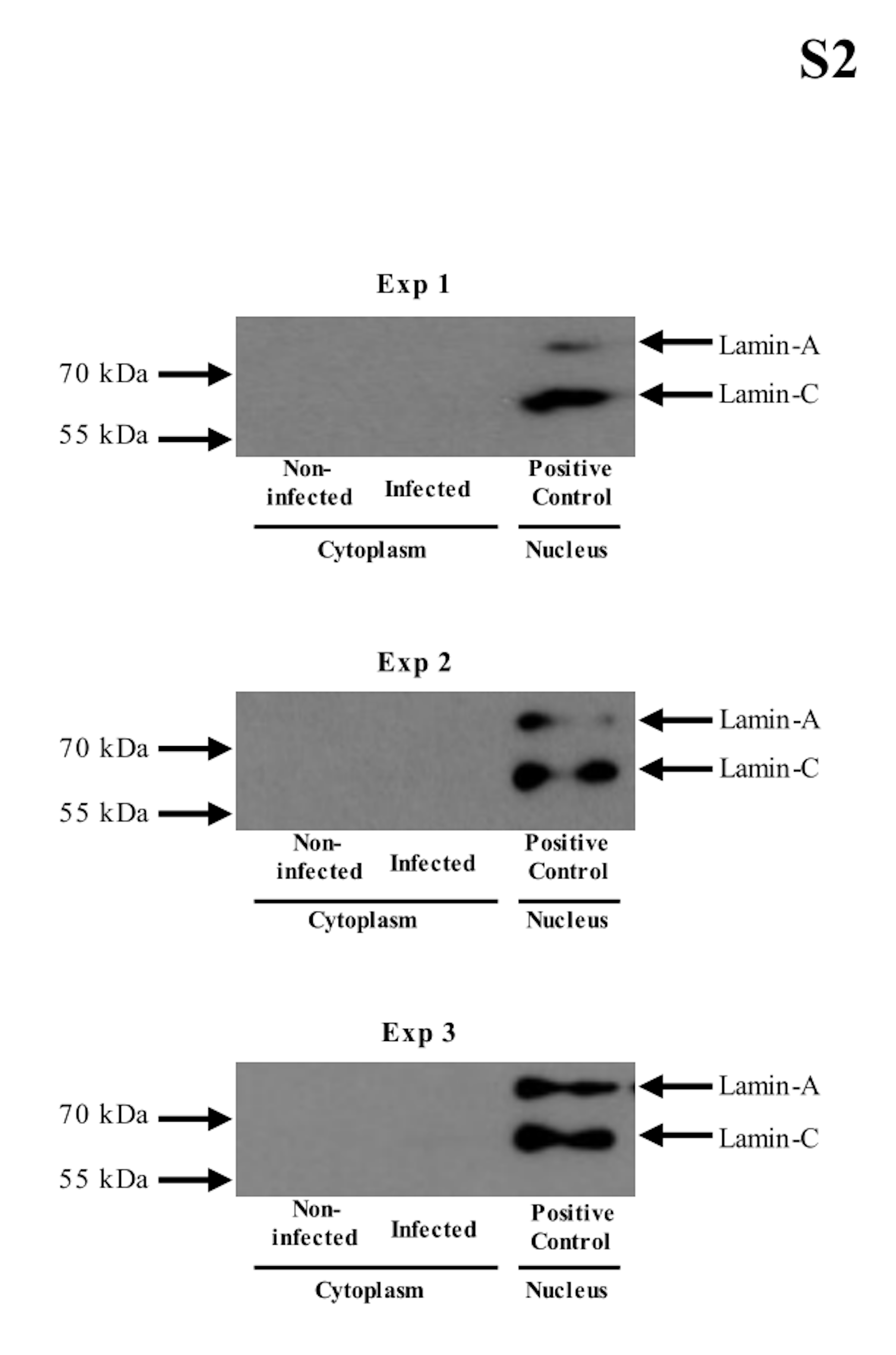

Supplement: Fig S2 — A) The cytoplasmic fraction of non-infected and Leishmania-infected dTHP-1 cells and the nuclear fraction of non-infected dTHP-1 cells were analyzed by Western blot for the nuclear marker Lamin A/C (n = 3). (TIFF) [file pone.0323227.s002.tiff]
