# Supplementary material for: Leishmania regulates host YY1: Comparative proteomic analysis identifies infection modulated YY1 dependent proteins
Source: PLoS One. 2025 May 15;20(5):e0323227. doi: 10.1371/journal.pone.0323227 (PMC12080872; doi:10.1371/journal.pone.0323227)
Supplement: File S1 — Table S4 represents biological process, Table S5 represents molecular function, and Table S6 represents cellular component. (DOCX) [file pone.0323227.s006.docx]

**Table S4: Gene ontology analysis (biological process) of significantly modulated proteins in YY1 knockdown dTHP-1 cells**

| **Proteins significantly modulated by YY1 knockdown in dTHP-1 cells (Biological Process)** | | | | |
| --- | --- | --- | --- | --- |
|  | **Upregulated proteins** | | **Downregulated proteins** | |
| **GO Term (GO ID)** | **Number of genes** | **Percentage** | **Number of genes** | **Percentage** |
| anatomical structure development (GO:0048856) | 94 | 32.41% | 79 | 33.19% |
| signaling (GO:0023052) | 86 | 29.66% | 71 | 29.83% |
| cell differentiation (GO:0030154) | 65 | 22.41% | 59 | 24.79 |
| regulation of DNA-templated transcription (GO:0006355) | 57 | 19.66% | 47 | 19.75 |
| immune system process (GO:0002376) | 44 | 15.17% | 48 | 20.17% |
| vesicle-mediated transport (GO:0016192) | 42 | 14.48% | 35 | 14.71% |
| programmed cell death (GO:0012501) | 37 | 12.76% | 25 | 10.50% |
| cell adhesion (GO:0007155) | 34 | 11.72% | 18 | 7.56% |
| transmembrane transport (GO:0055085) | 31 | 10.69% | 10 | 4.20% |
| cytoskeleton organization (GO:0007010) | 30 | 10.34% | 31 | 13.03 |
| protein-containing complex assembly (GO:0065003) | 30 | 10.34% | 28 | 11.76% |
| cell motility (GO:0048870) | 29 | 10.00% | 21 | 8.82% |
| carbohydrate derivative metabolic process (GO:1901135) | 26 | 8.97% | 18 | 7.56% |
| membrane organization (GO:0061024) | 26 | 8.97% | 13 | 5.46% |
| lipid metabolic process (GO:0006629) | 21 | 7.24% | 16 | 6.72% |
| protein catabolic process (GO:0030163) | 20 | 6.90% | 15 | 6.30% |
| mRNA metabolic process (GO:0016071) | 18 | 6.21% | 18 | 7.56% |
| protein maturation (GO:0051604) | 18 | 6.21% | 7 | 2.94% |
| reproductive process (GO:0022414) | 18 | 6.21% | 23 | 9.66% |
| defense response to other organism (GO:0098542) | 17 | 5.86% | 18 | 7.56% |
| telomere organization (GO:0032200) | 0 | 0 | 7 | 2.94% |
| intracellular protein transport (GO:0006886) | 16 | 5.52% | 13 | 5.46% |
| autophagy (GO:0006914) | 15 | 5.17% | 9 | 3.78% |
| Ribosome biogenesis (GO: 0042254) | 0 | 0 | 10 | 4.20% |
| chromatin organization (GO:0006325) | 15 | 5.17% | 14 | 5.88% |
| inflammatory response (GO:0006954) | 15 | 5.17% | 11 | 4.62% |
| mitotic cell cycle (GO:0000278) | 15 | 5.17% | 30 | 12.61% |
| generation of precursor metabolites and energy (GO:0006091) | 14 | 4.83% | 4 | 1.68% |
| cell junction organization (GO:0034330) | 13 | 4.48% | 8 | 3.36% |
| lysosome organization (GO:0007040) | 0 | 0 | 3 | 1.26% |
| DNA repair (GO:0006281) | 12 | 4.14% | 18 | 7.56% |
| mitochondrion organization (GO:0007005) | 12 | 4.14% | 7 | 2.94% |
| nucleobase-containing small molecule metabolic process (GO:0055086) | 12 | 4.14% | 15 | 6.30% |
| DNA-templated transcription (GO:0006351) | 11 | 3.79% | 9 | 3.78% |
| cilium organization (GO:0044782) | 0 | 0 | 4 | 1.68% |
| nervous system process (GO:0050877) | 11 | 3.79% | 10 | 4.20% |
| circulatory system process (GO:0003013) | 9 | 3.10% | 6 | 2.52% |
| amino acid metabolic process (GO:0006520) | 8 | 2.76% | 6 | 2.52% |
| establishment or maintenance of cell polarity (GO:0007163) | 8 | 2.76% | 9 | 3.78% |
| sulfur compound metabolic process (GO:0006790) | 8 | 2.76% | 8 | 3.36% |
| chromosome segregation (GO:0007059) | 6 | 2.07% | 18 | 7.56% |
| regulatory ncRNA-mediated gene silencing (GO:0031047) | 6 | 2.07% | 4 | 1.68% |
| wound healing (GO:0042060) | 6 | 2.07% | 6 | 2.52% |
| DNA replication (GO:0006260) | 5 | 1.72% | 7 | 2.94% |
| microtubule-based movement (GO:0007018) | 5 | 1.72% | 9 | 3.78% |
| muscle system process (GO:0003012) | 5 | 1.72% | 5 | 2.10% |
| protein folding (GO:0006457) | 5 | 1.72% | 0 | 0 |
| protein glycosylation (GO:0006486) | 5 | 1.72% | 5 | 2.10% |
| protein localization to plasma membrane (GO:0072659) | 5 | 1.72% | 4 | 1.68% |
| DNA recombination (GO:0006310) | 4 | 1.38% | 7 | 2.94% |
| extracellular matrix organization (GO:0030198) | 4 | 1.38% | 0 | 0 |
| modified amino acid metabolic process (GO:0006575) | 4 | 1.38% | 3 | 1.26% |
| cytokinesis (GO:0000910) | 3 | 1.03% | 4 | 1.68% |
| endocrine process (GO:0050886) | 3 | 1.03% | 0 | 0 |
| meiotic nuclear division (GO:0140013) | 3 | 1.03% | 0 | 0 |
| mitochondrial gene expression (GO:0140053) | 3 | 1.03% | 6 | 2.52% |
| nucleocytoplasmic transport (GO:0006913) | 3 | 1.03% | 7 | 2.94% |
| tRNA metabolic process (GO:0006399) | 3 | 1.03% | 9 | 3.78% |

**Table S5: Gene ontology analysis (molecular function) of significantly modulated proteins in YY1 knockdown dTHP-1 cells**

| **Proteins significantly modulated by YY1 knockdown in dTHP-1 cells (Molecular function)** | | | | |
| --- | --- | --- | --- | --- |
|  | **Upregulated proteins** | | **Downregulated proteins** | |
| **GO Term (GO ID)** | **Number of genes** | **Percentage** | **Number of genes** | **Percentage** |
| catalytic activity (GO:0003824) | 105 | 36.21% | 109 | 45.80% |
| hydrolase activity (GO:0016787) | 48 | 16.55% | 48 | 20.17% |
| catalytic activity, acting on a protein (GO:0140096) | 44 | 15.17% | 0 | 0 |
| DNA binding (GO:0003677) | 42 | 14.48% | 37 | 15.55% |
| molecular adaptor activity (GO:0060090) | 39 | 13.45% | 17 | 7.14% |
| RNA binding (GO:0003723) | 37 | 12.76% | 56 | 23.53% |
| transcription regulator activity (GO:0140110) | 36 | 12.41% | 15 | 6.30% |
| transferase activity (GO:0016740) | 35 | 12.07% | 47 | 19.75% |
| cytoskeletal protein binding (GO:0008092) | 23 | 7.93% | 25 | 10.50% |
| molecular function regulator activity (GO:0098772) | 23 | 7.93% | 23 | 9.66% |
| oxidoreductase activity (GO:0016491) | 23 | 7.93% | 10 | 4.20% |
| transporter activity (GO:0005215) | 21 | 7.24% | 8 | 3.36% |
| lipid binding (GO:0008289) | 14 | 4.83% | 14 | 5.88% |
| ATP-dependent activity (GO:0140657) | 13 | 4.48% | 17 | 7.14% |
| molecular transducer activity (GO:0060089) | 12 | 4.14% | 5 | 2.10% |
| structural molecule activity (GO:0005198) | 12 | 4.14% | 8 | 3.36% |
| catalytic activity, acting on RNA (GO:0140098) | 10 | 3.45% | 0 | 0 |
| GTPase activity (GO:0003924) | 6 | 2.07% | 8 | 3.36% |
| catalytic activity, acting on DNA (GO:0140097) | 5 | 1.72% | 0 | 0 |
| histone binding (GO:0042393) | 5 | 1.72% | 5 | 2.10% |
| ligase activity (GO:0016874) | 4 | 1.38% | 8 | 3.36% |
| translation regulator activity (GO:0045182) | 4 | 1.38% | 3 | 1.26% |
| virus receptor activity (GO:0001618) | 0 | 0 | 3 | 1.26% |
| cargo receptor activity (GO:0038024) | 3 | 1.03% | 0 | 0 |
| isomerase activity (GO:0016853) | 0 | 0 | 3 | 1.26% |

**Table S6: Gene ontology analysis (cellular component) of significantly modulated proteins in YY1 knockdown dTHP-1 cells**

| **Proteins significantly modulated by YY1 knockdown in dTHP-1 cells (Cellular Component)** | | | | |
| --- | --- | --- | --- | --- |
|  | **Upregulated proteins** | | **Downregulated proteins** | |
| **GO Term (GO ID)** | **Number of genes** | **Percentage** | **Number of genes** | **Percentage** |
| nucleus ( GO:0005634 ) | 142 | 48.97% | 131 | 55.04% |
| cytosol ( GO:0005829 ) | 115 | 39.66% | 124 | 52.10% |
| nucleoplasm (GO:0005654) | 86 | 29.66% | 94 | 39.50% |
| plasma membrane (GO:0005886) | 82 | 28.28% | 57 | 23.95% |
| extracellular region (GO:0005576) | 71 | 24.48% | 51 | 21.43% |
| cytoplasmic vesicle (GO:0031410) | 56 | 19.31% | 48 | 20.17% |
| mitochondrion (GO:0005739) | 49 | 16.90% | 26 | 10.92% |
| endoplasmic reticulum (GO:0005783) | 42 | 14.48% | 22 | 9.24% |
| Golgi apparatus (GO:0005794) | 37 | 12.76% | 32 | 13.45% |
| cytoskeleton (GO:0005856) | 34 | 11.72% | 41 | 17.23% |
| chromosome (GO:0005694) | 30 | 10.34% | 37 | 15.55% |
| vacuole (GO:0005773) | 24 | 8.28% | 15 | 6.30% |
| lysosome (GO:0005764) | 23 | 7.93% | 13 | 5.46% |
| endosome (GO:0005768) | 22 | 7.59% | 25 | 10.50% |
| nucleolus (GO:0005730) | 20 | 6.90% | 25 | 10.50% |
| microtubule organizing center (GO:0005815) | 10 | 3.45% | 14 | 5.88% |
| nuclear envelope (GO:0005635) | 9 | 3.10% | 9 | 3.78% |
| external encapsulating structure (GO:0030312) | 6 | 2.07% | 0 | 0 |
| extracellular matrix (GO:0031012) | 6 | 2.07% | 0 | 0 |
| lipid droplet (GO:0005811) | 4 | 1.38% | 3 | 1.26% |
| cilium (GO:0005929) | 3 | 1.03% | 4 | 1.68% |
| [peroxisome (GO:0005777)](http://amigo.geneontology.org/amigo/term/GO:0005777) | 0 | 0 | 4 | 1.68% |
| [ribosome (GO:0005840)](http://amigo.geneontology.org/amigo/term/GO:0005840) | 0 | 0 | 4 | 1.68% |
