# Supplementary material for: Leishmania regulates host YY1: Comparative proteomic analysis identifies infection modulated YY1 dependent proteins
Source: PLoS One. 2025 May 15;20(5):e0323227. doi: 10.1371/journal.pone.0323227 (PMC12080872; doi:10.1371/journal.pone.0323227)
Supplement: S1 File — (PDF) [file pone.0323227.s007.pdf]

Fig 1A. Western Blot of YY1 knockdown using three unique siRNAs or scrambled RNA in dTHP-1 cells

10% SDS-PAGE
Nitrocellulose

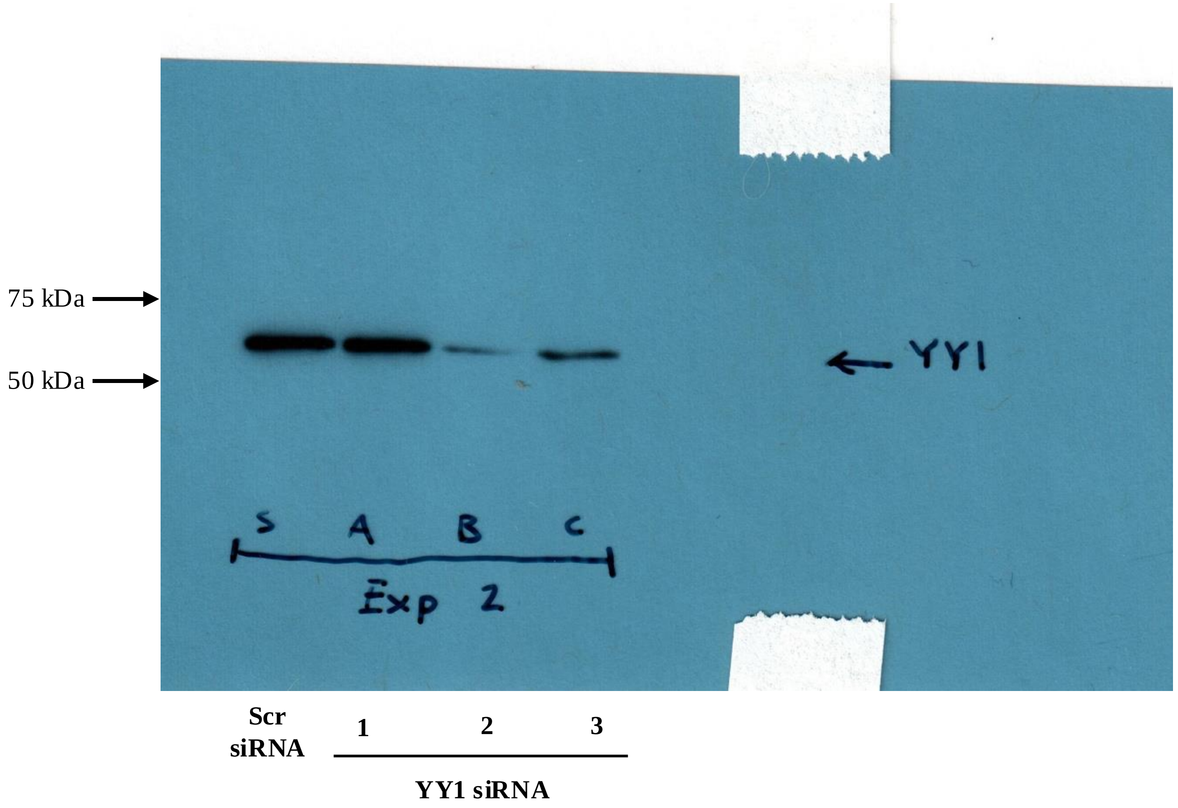

Image captured via ECL and X-ray film

Fig 1A. Western Blot of YY1 knockdown using three unique siRNAs or scrambled RNA in dTHP-1 cells

10% SDS-PAGE
Nitrocellulose

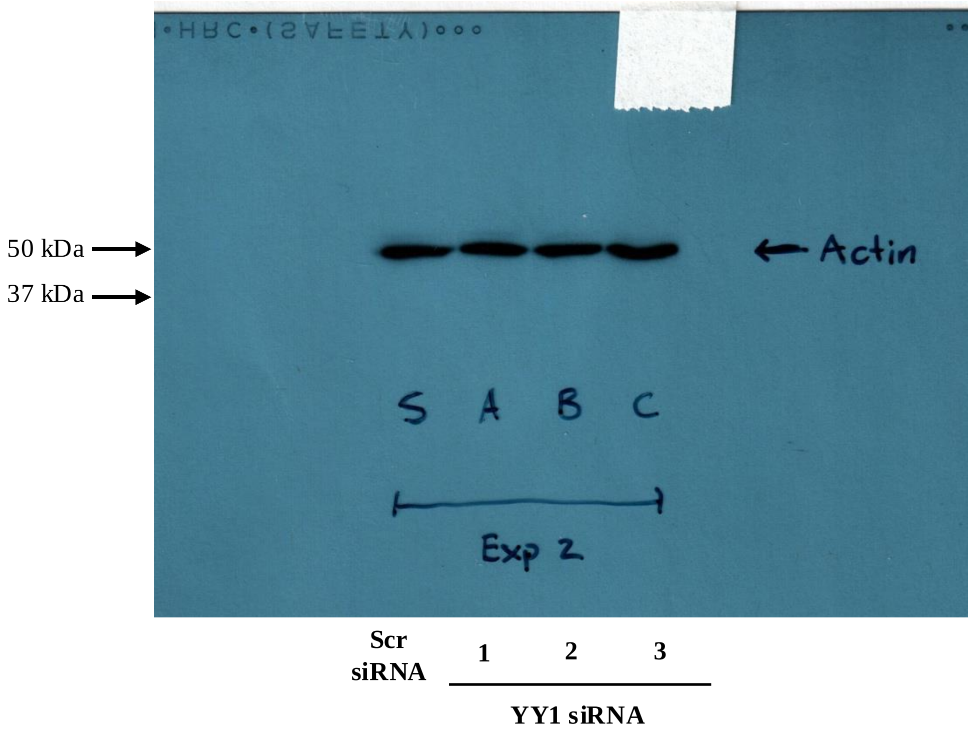

Image captured via ECL and X-ray film

Fig 2B. Western Blot of YY1 knockdown using two unique siRNAs or scrambled RNA in hMDMs

10% SDS-PAGE
Nitrocellulose

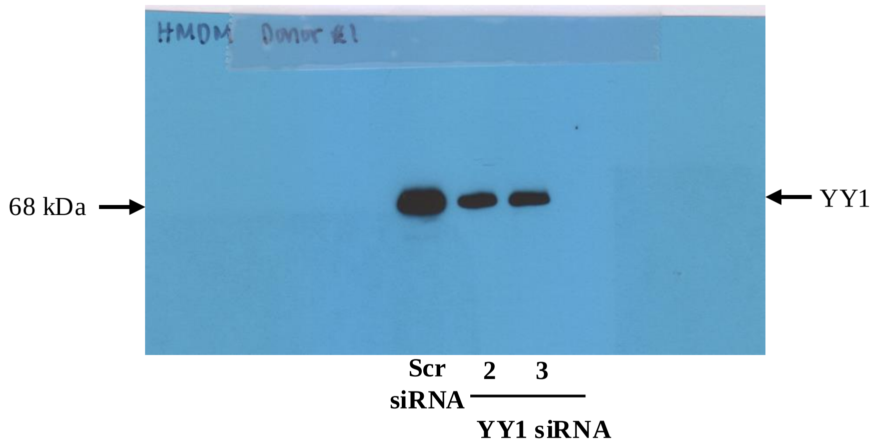

Image captured via ECL and X-ray film

Fig 2B. Western Blot of YY1 knockdown using two unique siRNAs or scrambled RNA in hMDMs

10% SDS-PAGE
Nitrocellulose

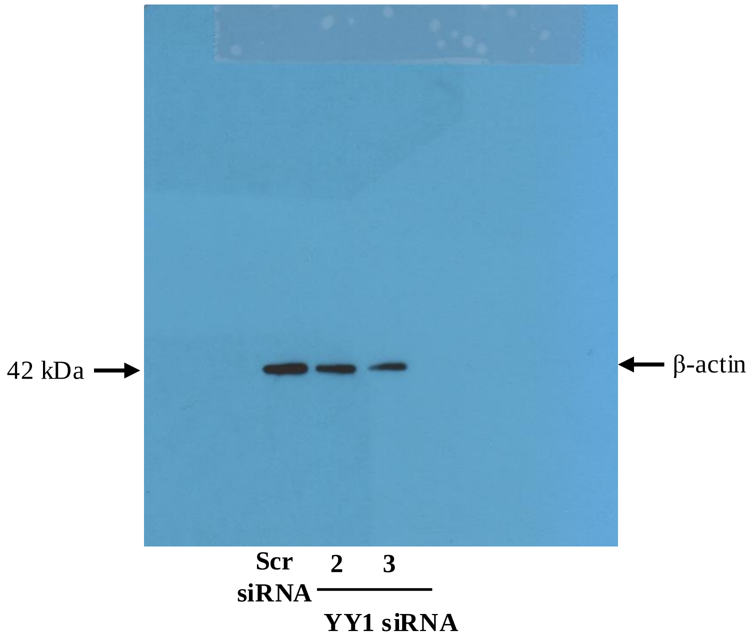

Image captured via ECL and X-ray film

Fig 3A & C Western Blot of cytoplasmic and nuclear YY1 expression in non-infected and infected dTHP-1 cells

10% SDS-PAGE
Nitrocellulose

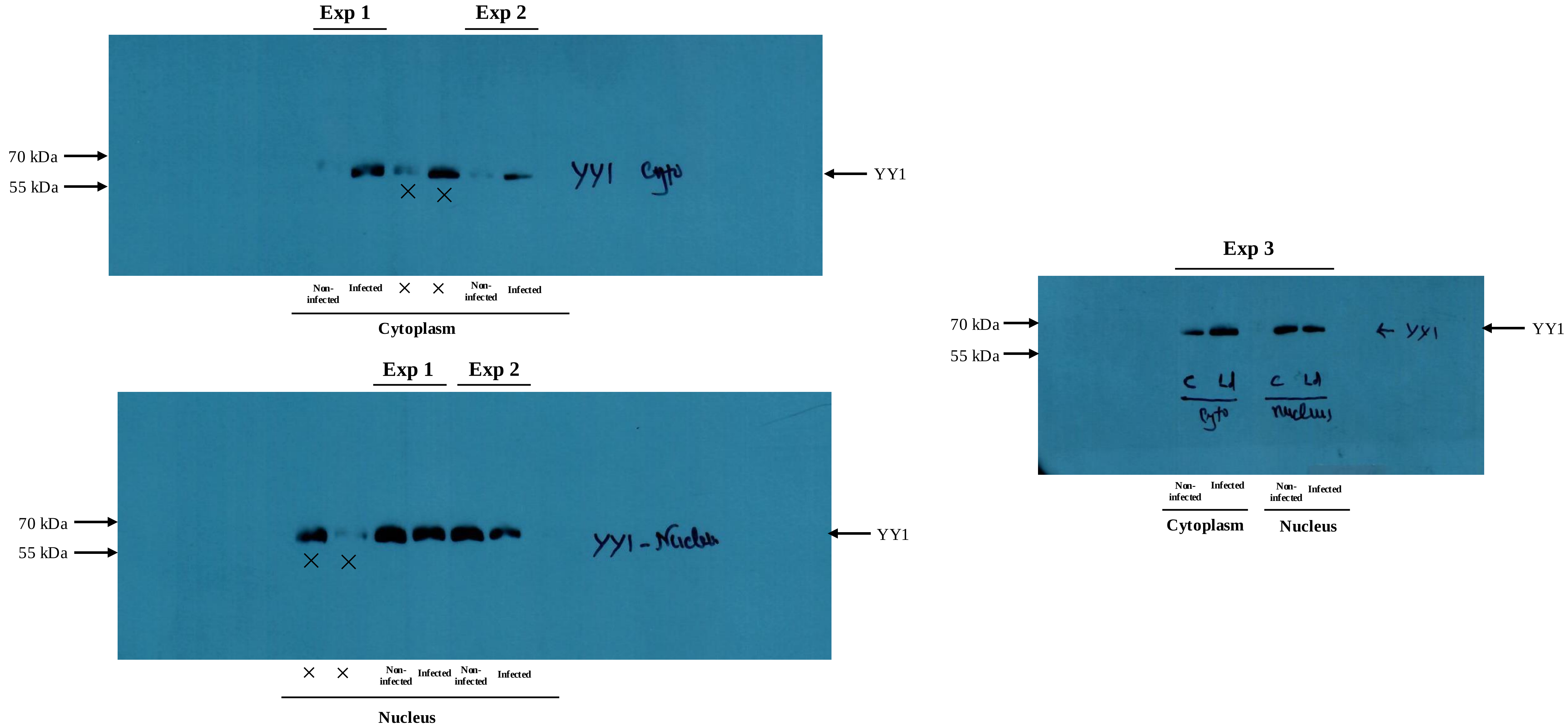

Image captured via ECL and X-ray film

Fig 3A & C Western Blot of Lamin A and C in nuclear fraction of non-infected and infected dTHP-1 cells

10% SDS-PAGE
Nitrocellulose

Non-infected Infected Non-infected

Nucleus

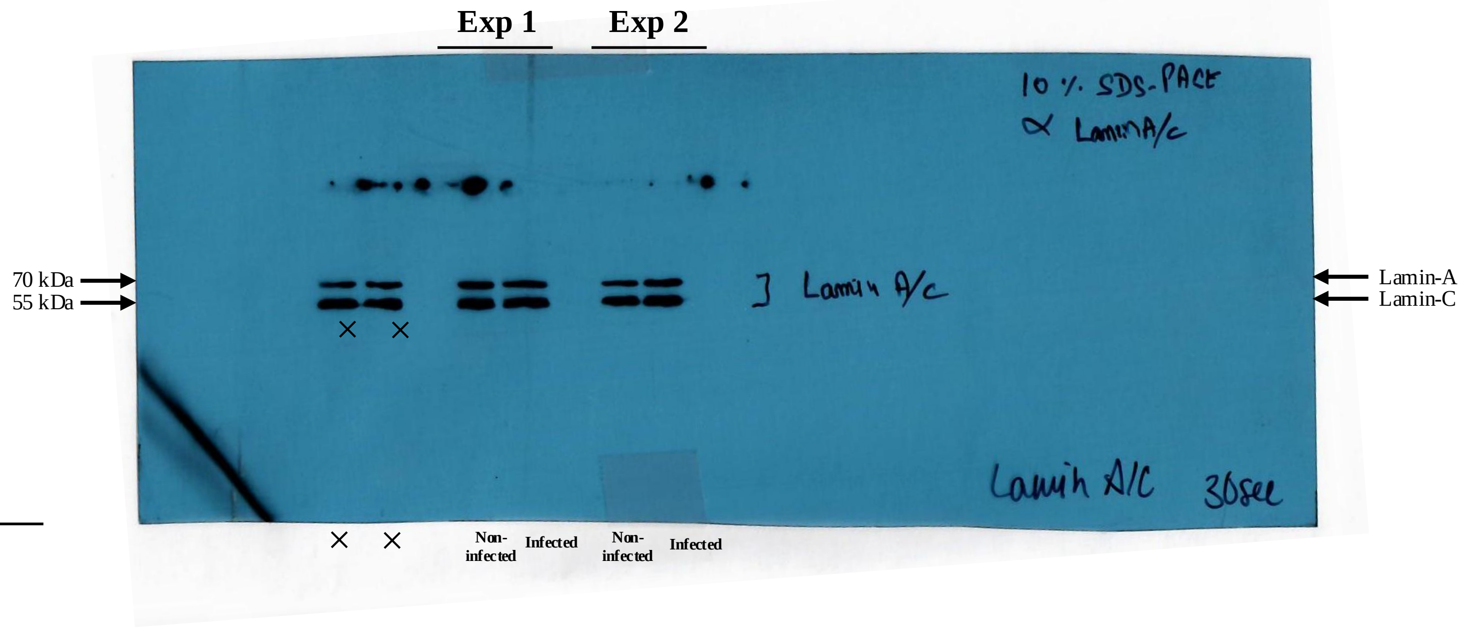

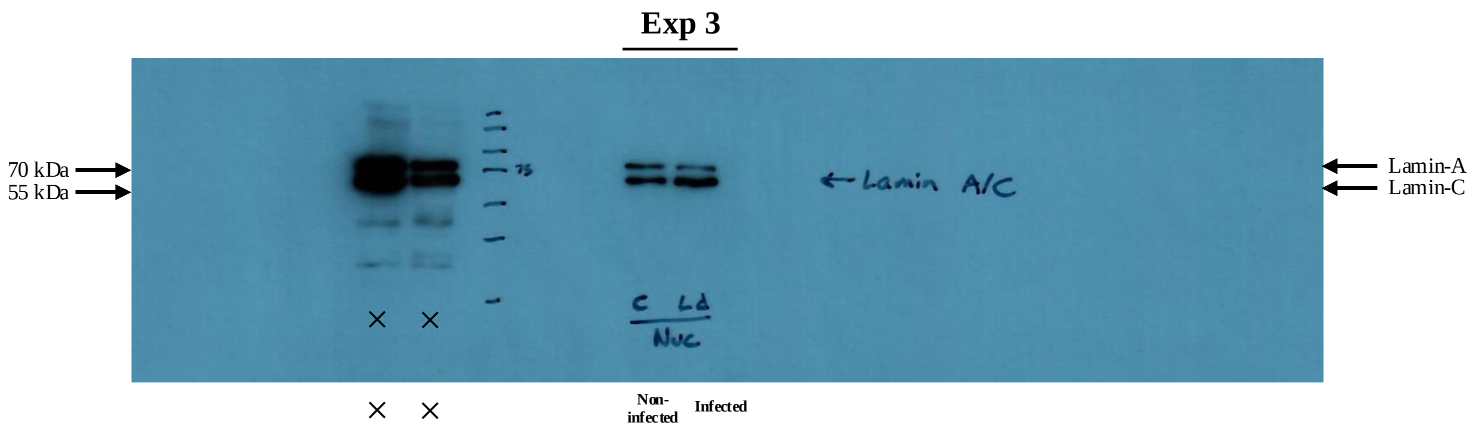

Image captured via ECL and X-ray film

Fig 3A & C Western Blot of GAPDH expression in non-infected and infected dTHP-1 cells

11% SDS-PAGE
Nitrocellulose

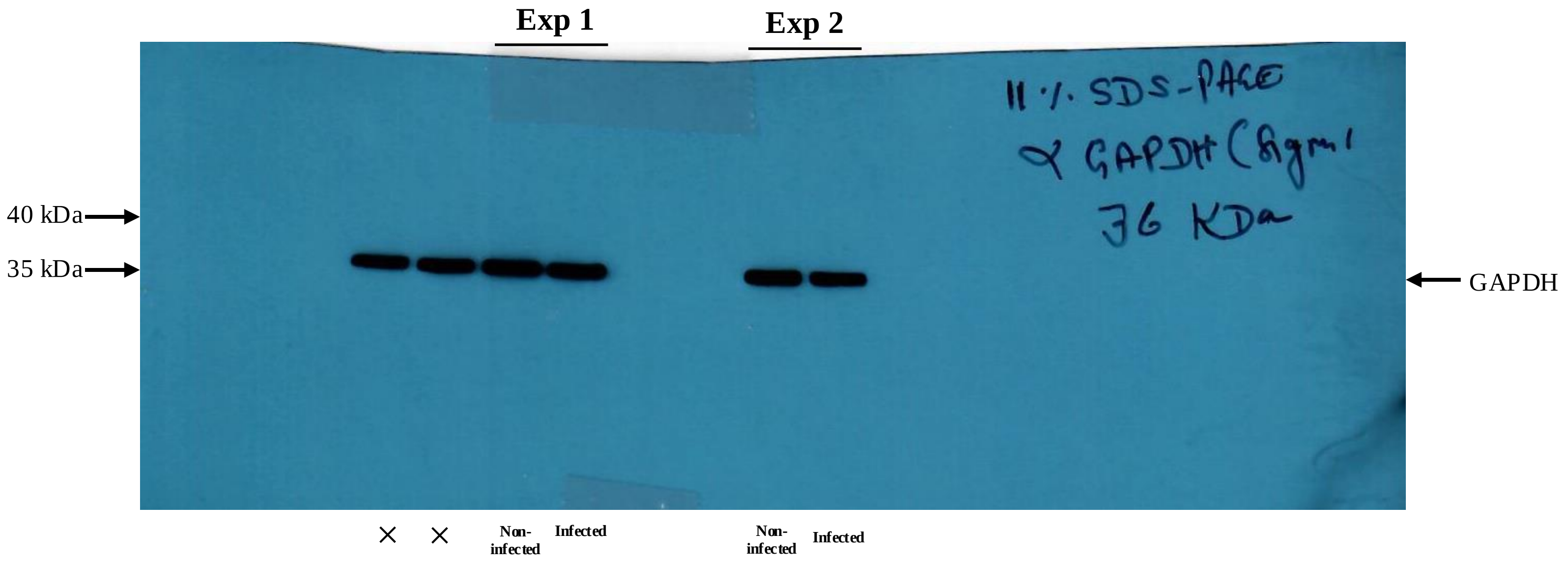

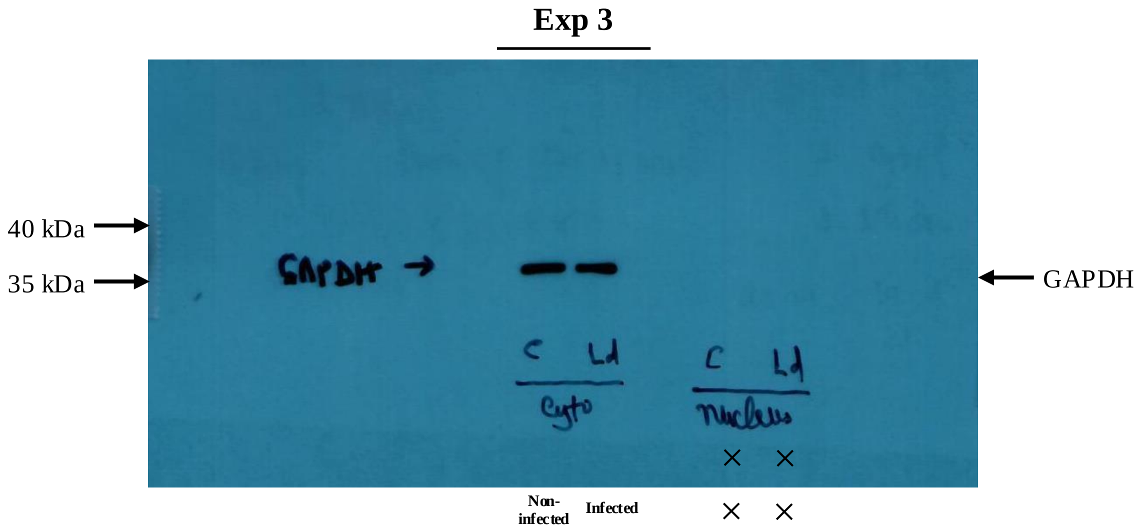

Image captured via ECL and X-ray film

Fig 4A. Western Blot of YY1 expression in cytoplasmic fractions from dTHP-1 cells after leptomycin treatment

10% SDS-PAGE
Nitrocellulose

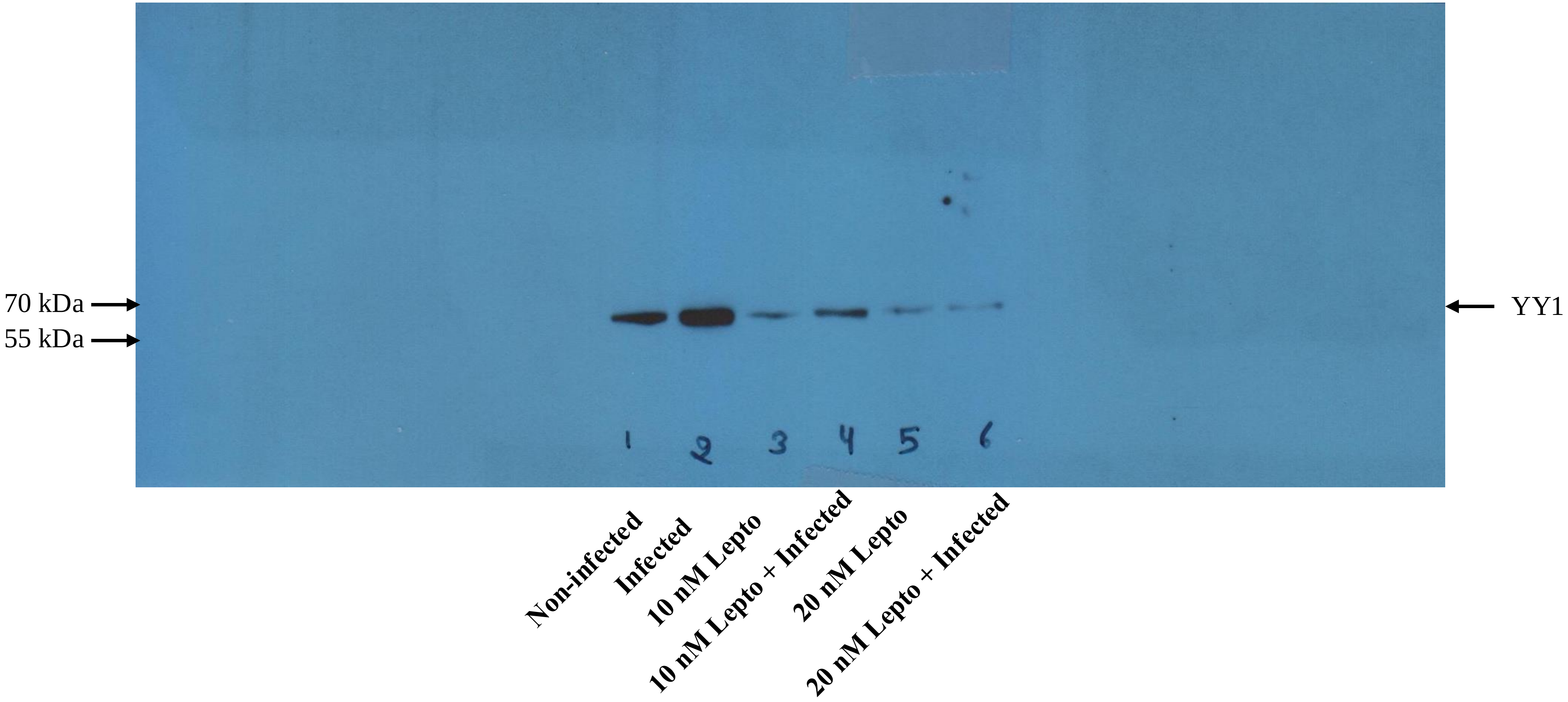

Image captured via ECL and X-ray film

Fig 4A. Western Blot of GAPDH in cytoplasmic fractions from dTHP-1 cells after leptomycin treatment

11% SDS-PAGE
Nitrocellulose

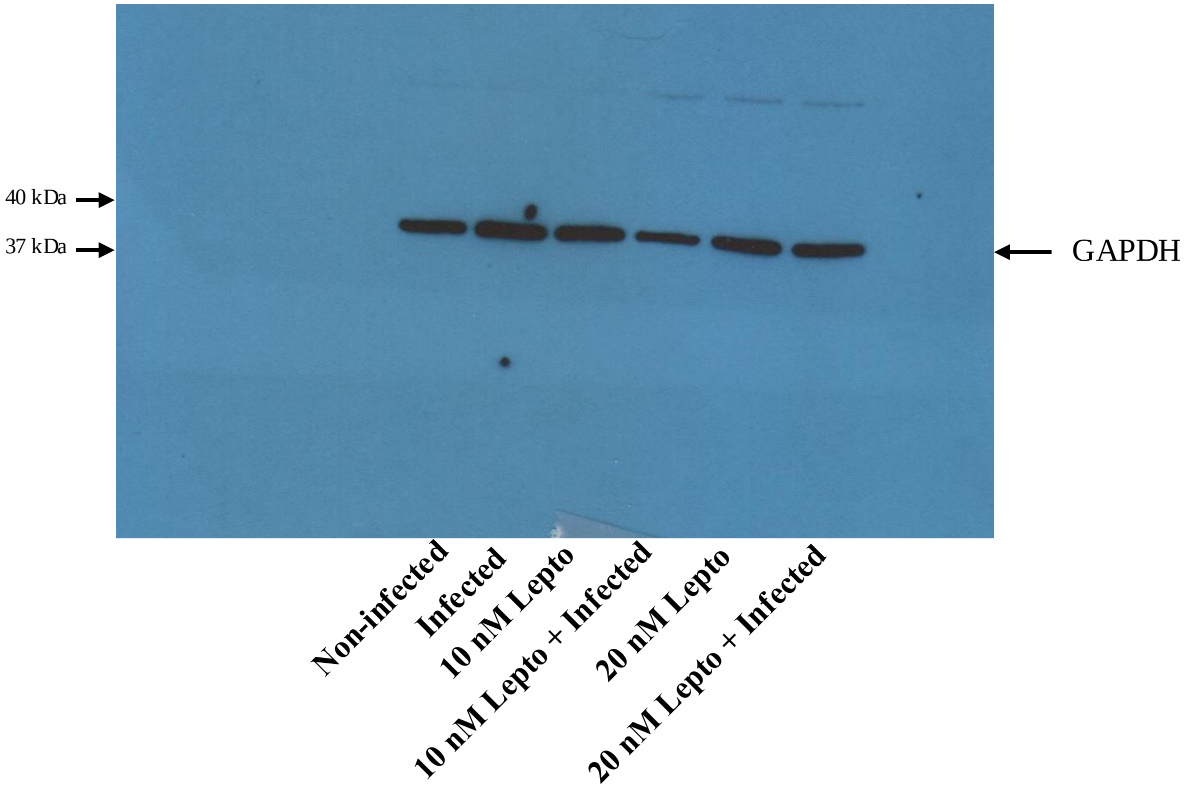

Image captured via ECL and X-ray film

Fig S1A Western Blot of YY1 expression in whole cell lysates of infected dTHP-1 at different MOI and different time points

10% SDS-PAGE

Nitrocellulose

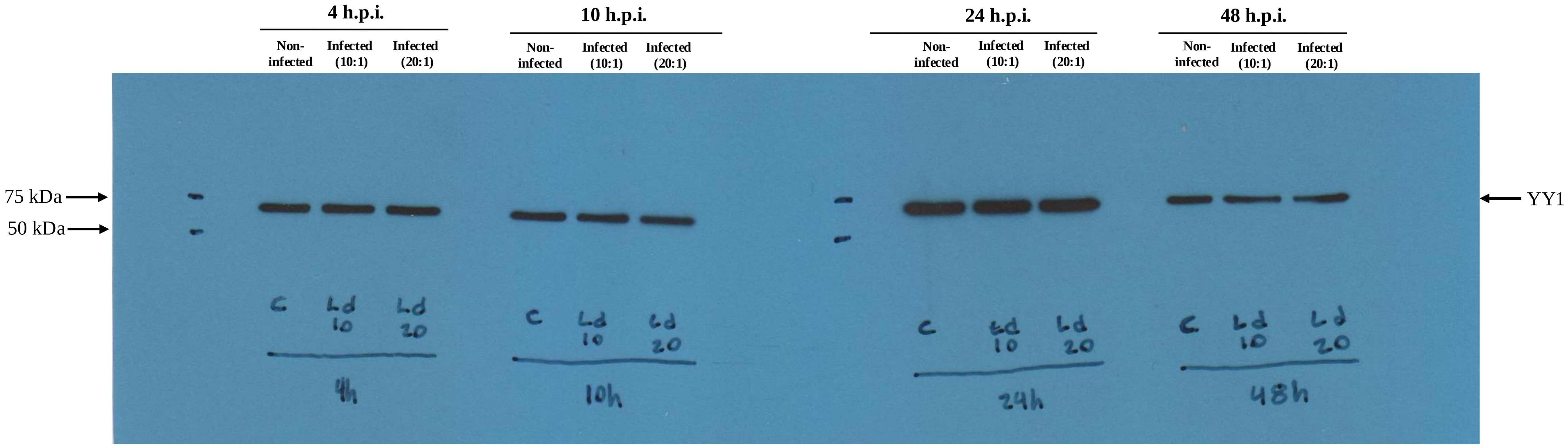

Image captured via ECL and X-ray film

Fig S1A Western Blot of YY1 expression in whole cell lysates of infected dTHP-1 at different MOI and different time points

10% SDS-PAGE
Nitrocellulose

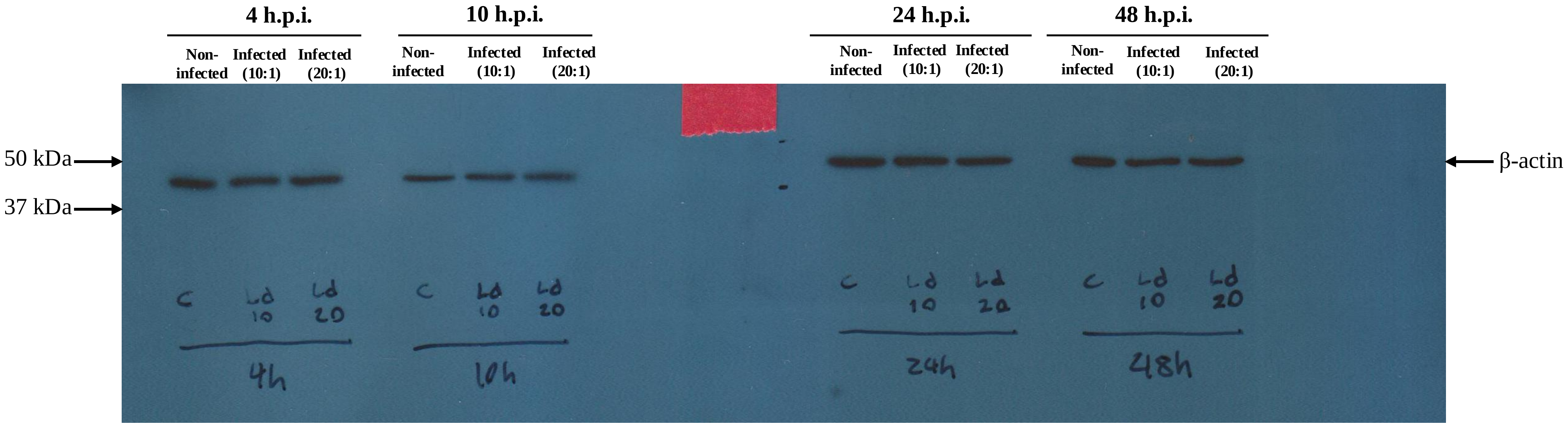

Image captured via ECL and X-ray film

Fig S2. Western Blot to check comtanimation of lamin a/c in cytosol of dTHP-1 cells

10% SDS-PAGE

Nitrocellulose

Exp 1

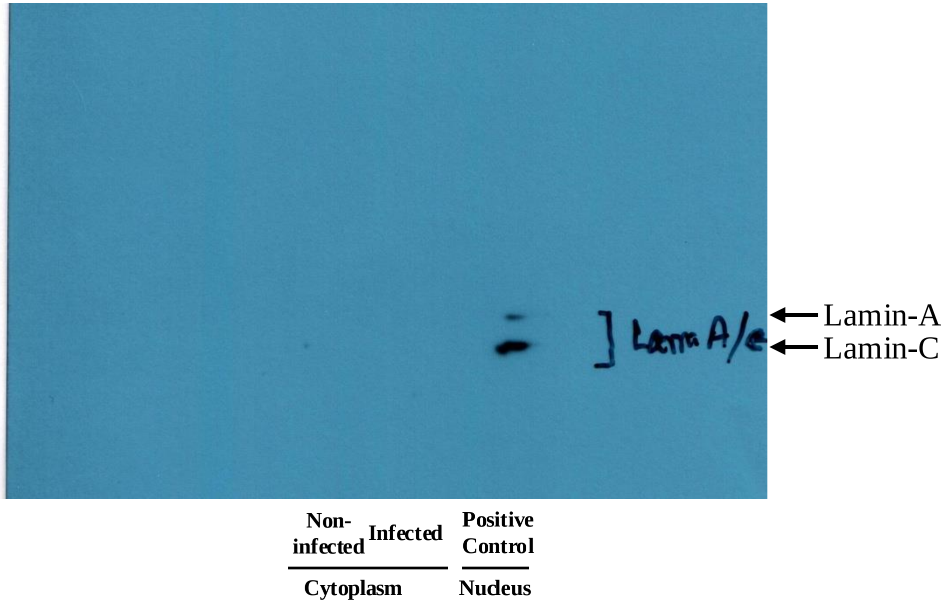

Exp 2

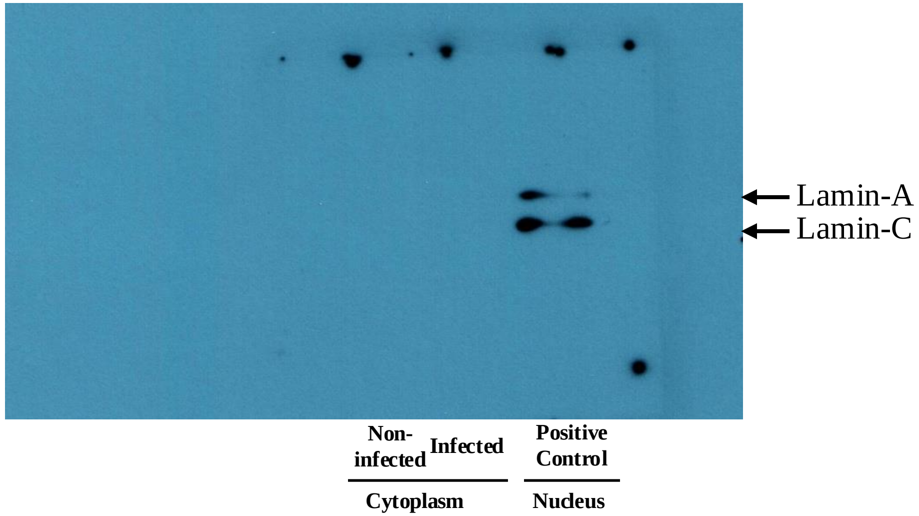

Exp 3

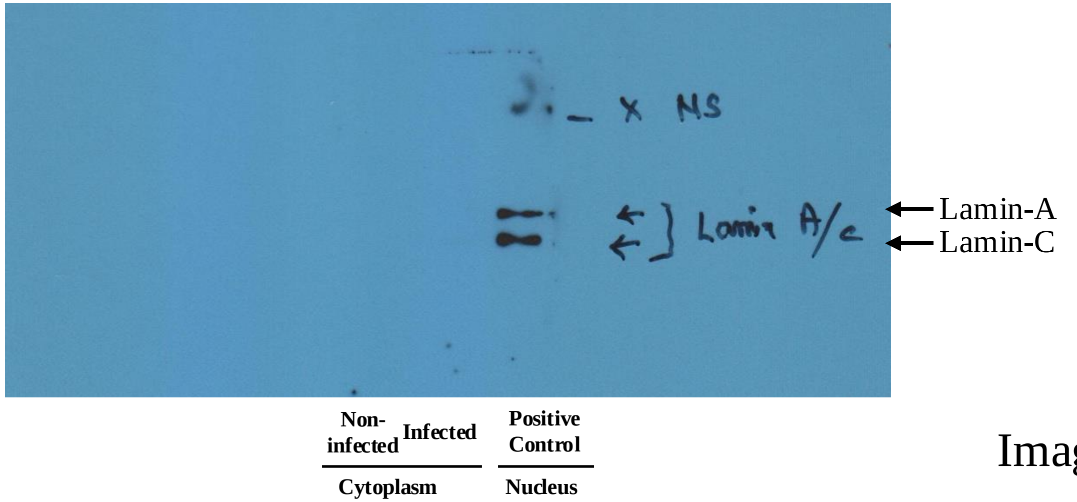

Image captured via ECL and X-ray film
